# Liver in infections: a single-cell and spatial transcriptomics perspective

**DOI:** 10.1186/s12929-023-00945-z

**Published:** 2023-07-10

**Authors:** Ju Zou, Jie Li, Xiao Zhong, Daolin Tang, Xuegong Fan, Ruochan Chen

**Affiliations:** 1 Hunan Key Laboratory of Viral Hepatitis, Xiangya Hospital, Central South University, Changsha, 410008 Hunan China; 2 Department of Infectious Diseases, Xiangya Hospital, Central South University, Changsha, 410008 Hunan China; 3 Department of Surgery, UT Southwestern Medical Center, Dallas, TX USA

**Keywords:** Liver, Infections, Single-cell technologies, Spatial transcriptome

## Abstract

The liver is an immune organ that plays a vital role in the detection, capture, and clearance of pathogens and foreign antigens that invade the human body. During acute and chronic infections, the liver transforms from a tolerant to an active immune state. The defence mechanism of the liver mainly depends on a complicated network of intrahepatic and translocated immune cells and non-immune cells. Therefore, a comprehensive liver cell atlas in both healthy and diseased states is needed for new therapeutic target development and disease intervention improvement. With the development of high-throughput single-cell technology, we can now decipher heterogeneity, differentiation, and intercellular communication at the single-cell level in sophisticated organs and complicated diseases. In this concise review, we aimed to summarise the advancement of emerging high-throughput single-cell technologies and re-define our understanding of liver function towards infections, including hepatitis B virus, hepatitis C virus, *Plasmodium*, schistosomiasis, endotoxemia, and corona virus disease 2019 (COVID-19). We also unravel previously unknown pathogenic pathways and disease mechanisms for the development of new therapeutic targets. As high-throughput single-cell technologies mature, their integration into spatial transcriptomics, multiomics, and clinical data analysis will aid in patient stratification and in developing effective treatment plans for patients with or without liver injury due to infectious diseases.

**Supplementary Information:**

The online version contains supplementary material available at 10.1186/s12929-023-00945-z.

## Introduction

**Fig. 1:**
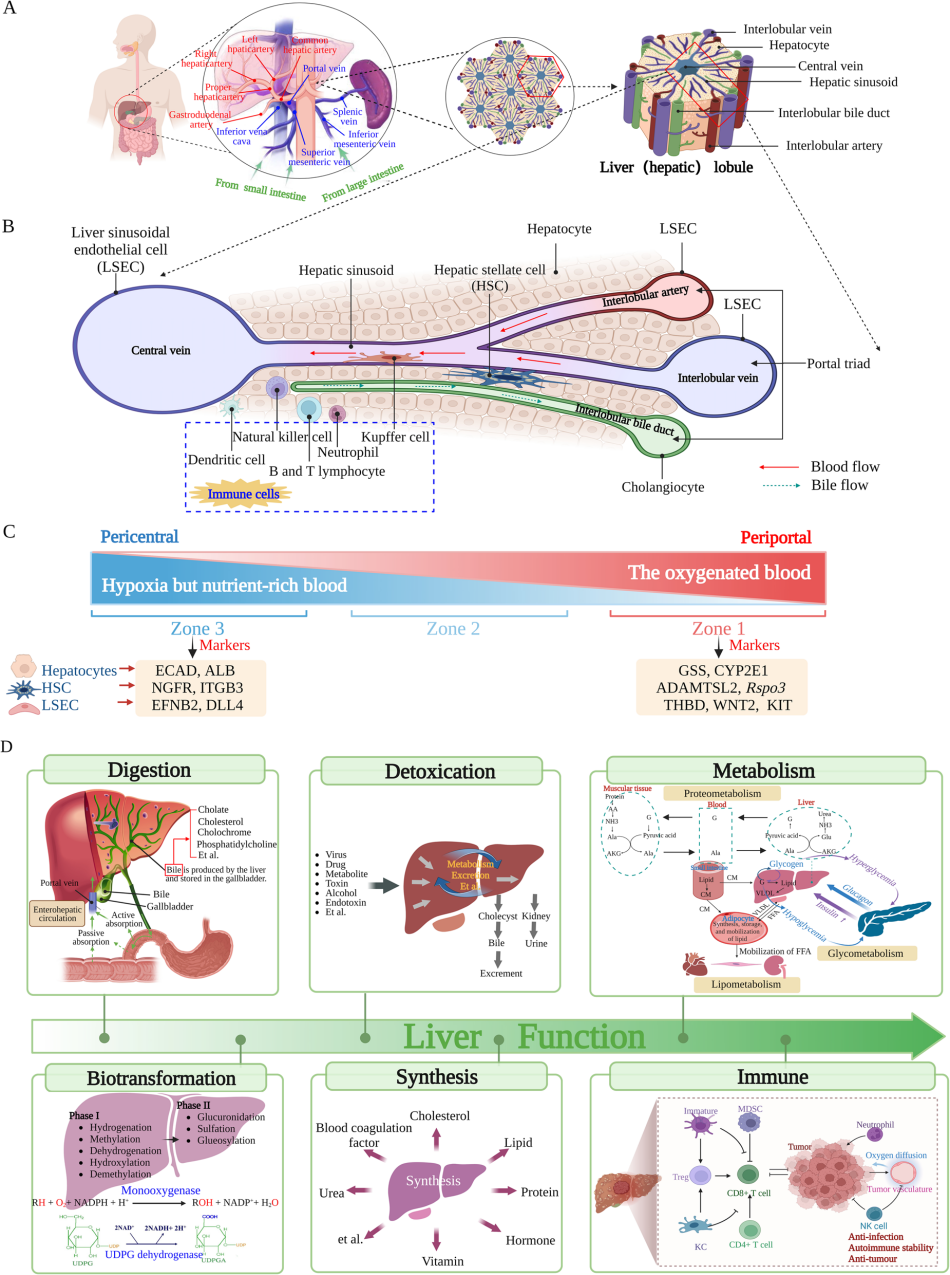
Anatomical structure, composition, and function of the liver. **A** Arterial supply to the liver and portal venous drainage. **B** Anatomical structure and cellular composition of hepatic lobules. **C** Single-cell RNA sequencing has revealed gradients of gene expression along the three different zones of hepatic lobule in different cell lineages, enabling the differentiation of portal zone and central zone hepatocytes, LSECs, and HSCs. These markers are shown in Figure. **D** Main functions of the liver. *ALB* albumin, *ADAMTSL2* ADAMTS-like protein 2, *CYP2E1* cytochrome P450 2E1, *DLL4* Delta-like protein 4, *ECAD* E-cadherin, *EFNB2* ephrin-B2, *GSS* glutathione synthetase, *HSC* hepatic stellate cell, *ITGB3* integrin β3, *KIT* mast/stem cell growth factor receptor Kit, *LSEC* liver sinusoidal endothelial cell, *NGFR* tumour necrosis factor receptor superfamily member 16, *Rspo3* R-spondin-3, *THBD* thrombomodulin, *WNT2* wingless-type MMTV integration site family member 2

The mammalian liver is a complex organ consisting of diverse cell types that perform multiple physiological functions including digestion, synthesis, metabolism, and detoxification. It also acts as an immune organ and plays a crucial role in anti-infection, autoimmune stability, and anti-tumour effects. In particular, the liver has a unique immunological advantage as its parenchymal and nonparenchymal cells can exert immune functions to participate in immunoregulation for maintaining homeostasis. As a fundamental immune organ, the liver receives blood directly through the portal vein draining from the peritoneum, gastrointestinal tract, pancreas, and spleen, while several classic immune sentinel surveillance sites, such as the lymph nodes and spleen, are omitted [1] (Fig. 1A). Under physiological circumstances, the liver is more heavily exposed to microorganisms and endotoxins than other organs or tissues owing to its unique anatomical location. Therefore, the liver usually maintains a tolerogenic immune state to prevent needless immune activation and excessive autoimmune responses [2, 3]. However, liver tolerance also allows viruses, parasites, and other microorganisms to persist chronically and cause long-term damage [4, 5]. The maintenance of the immunologically balanced state in the liver is essential for human health and survival. During acute and chronic infections, the liver transforms from a tolerant state to an active immune state and constitutes an important line of defence against invading microorganisms [6–9]. The defence mechanism of the liver depends mainly on a complex network of immune cells, including Kupffer cells (KCs), dendritic cells (DCs), neutrophils, natural killer (NK) cells, and B and T lymphocytes [10] (Fig. 1B). Furthermore, non-immune cells such as hepatocytes, choanocytes, hepatic stellate cells (HSCs), and liver sinusoidal endothelial cells (LSECs) contribute to immunity by recruiting proinflammatory immune cells (Fig. 1B) and simultaneously producing inflammatory cytokines such as acute-phase proteins, complement factors, and cell adhesion molecules [11–13]. Owing to the crucial role played by the liver in defence against harmful microorganisms, a comprehensive liver cell atlas in both healthy and diseased states is needed for new therapeutic target development and disease intervention improvement.

**Fig. 2:**
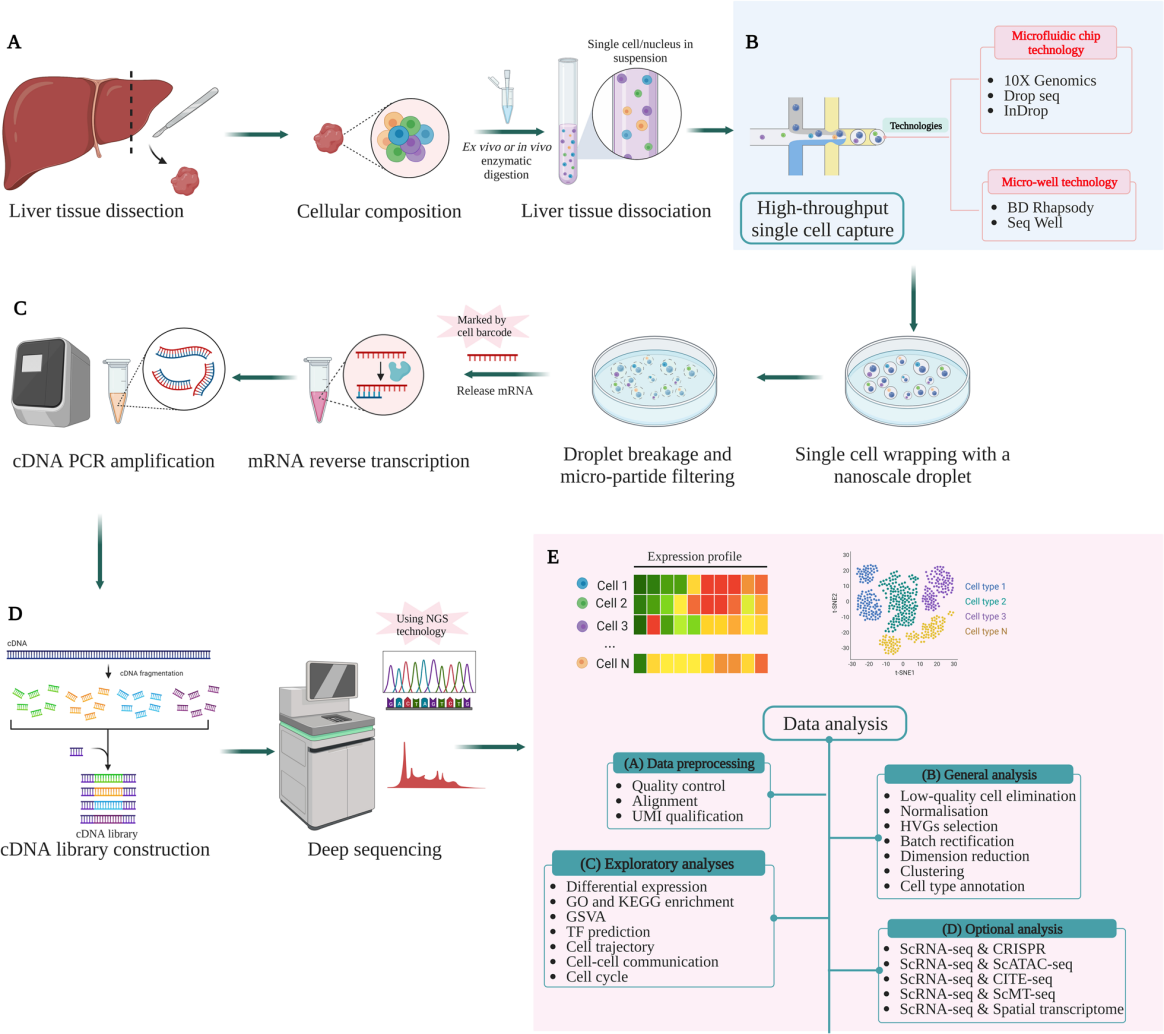
A concise overview of scRNA-seq workflow. *CRISPR* clustered regularly interspaced short palindromic repeats, *CITE-seq* cellular indexing of transcriptomes and epitopes by sequencing, *GSVA* gene set variation analysis, *HVGs* highly variable genes, *PCR* polymerase chain reaction, *ScRNA-seq* single-cell RNA sequencing, *ScATAC-seq* single-cell assay for transposase-accessible chromatin using sequencing, *ScMT-seq* single-cell methylome and transcriptome sequencing, *UMI* unique molecular identifier

Conventionally, the liver landscape in a healthy or diseased state is studied using whole-tissue sequencing of a bulk and mixed population of cells within different zonations, which makes it difficult to interpret the uniqueness of individual cells and cell–cell crosstalk in maintaining cellular homeostasis [14]. With the development of high-throughput single-cell technology, we can now decipher heterogeneity, differentiation, and intercellular communication at the single-cell level in sophisticated organs and complicated diseases. For example, single-cell RNA sequencing (scRNA-seq) can characterise the complete transcriptome of a single cell on a massive scale. This novel technology usually involves tissue dissociation, single-cell capture, cell lysis, and messenger ribonucleic acid (mRNA) marked by cell barcodes (Fig. 2A, C). Following RNA capture, the next steps are reverse transcription and amplification, complementary deoxyribonucleic acid (cDNA) library construction, and sequencing using next-generation sequencing technology (Fig. 2C, D). Using the aforementioned cell barcode, the sequence reads are then navigated back to the original cells, producing complete transcriptome data for thousands of cells in a single experiment (Fig. 2). In many studies, scRNA-seq has been used for better understanding of cellular and pathological mechanisms and gaining novel cellular and biomedical insights into disease pathology [15, 16].

To maintain liver homeostasis in a healthy state while remaining responsive to exogenous and endogenous stress, a highly organised and specialised structure of liver cells, including hormones, cytokines, metabolites, and microbial products, is needed to integrate local information at the molecular level. Liver research has embraced and benefited from innovative approaches (high-throughput single-cell technologies and multiomics), with several liver scRNA-seq studies being published in the past 5 years [17–23]. Recently, several reviews have summarised the application of single-cell techniques in various types of liver diseases, especially liver cancer [24], non-alcoholic fatty liver disease (NAFLD) [25], and fibrosis [6]. In our review, we elaborate on how emerging single-cell technologies strengthen our understanding of liver function during local and systemic infection, including the core tips of application schema in the liver, the single-cell atlas of the normal liver and diseased liver towards infection, clinical implications, and future directions. This concise review summarises recent findings and highlights new directions for the application of high-throughput single-cell and spatial technologies with multiomics to unravel previously unknown mechanisms for the advancement of new therapeutics targeting liver pathology in response to infections.

## Technology

### Advantages and disadvantages of scRNA-seq in liver research

RNA sequencing is increasingly being used to investigate phenotypes and deep driving mechanisms of liver pathologies. Whole-tissue RNA sequencing, also known as “bulk sequencing”, has been extensively used to identify major differences in the transcriptome between normal and diseased conditions [26, 27]. Bulk sequencing provides an average value of the molecular signal of every sample, which represents mixed RNA contents from different cells located within the same sample and is hence remarkably affected by cell type prevalence [28]. Therefore, bulk sequencing cannot be applied to investigate cell heterogeneity (i.e., cell subgroups among major cell types), specific pathogenic cell culprits, and rare cell subpopulations or to dissect tumour clonal evolution and the microenvironment.

Owing to its high accuracy and specificity, scRNA-seq has become an ideal research tool for single cell study [29]. Unbiased high-throughput research can be performed with a minimum starting sample volume. ScRNA-seq can be used alongside multiomics to simultaneously analyse the genome, transcriptome, epigenome, proteome, and metabolome [30]. The functional state of a single cell can be evaluated to identify and discover novel cell types in an unbiased manner. It can also be used to construct the differentiation track of the cell lineage and create a molecular map of the cell development lineage. Compared to traditional sequencing, scRNA-seq displays stronger discovery ability in new gene detection without knowing sequence information in advance and has higher sensitivity in rare mutations and transcript quantification [31]. Moreover, scRNA-seq avoids polymerase chain reaction (PCR) amplification bias. PCR amplification is required for sequencing in many cases; however, the limitation of amplification is that different nucleic acid sequences have different amplification degrees under the same conditions. Therefore, the abundance of nucleic acid sequences after amplification differs from that before amplification. ScRNA-seq can avoid the PCR amplification preference because it calculates the actual value of the nucleic acid sequence before amplification rather than the relative abundance of the nucleic acid sequence after amplification.

However, scRNA-seq has several limitations. First, the need for cell viability and quality is high and essential for subsequent analysis, whereas obtaining qualified single-cell suspensions is sometimes difficult. ScRNA-seq has some inherent methodological defects, one of which is “artificial transcriptional stress responses” where tissue dissociation could induce the expression of stress genes, resulting in artificial changes in cellular transcriptome patterns, as confirmed by several experiments [32, 33]. Thus, the dissociation process should be carefully optimised to achieve maximum production without inducing biases [32]. Moreover, the dynamic nature of gene expression can lead to noise in scRNA-seq technologies, as the levels of multiple gene transcripts are typically in a constant state of flux [34].

Secondly, it should be noted that scRNA-seq is limited to detecting the expression at the RNA level, and cannot provide information on protein expression [35]. The conversion from RNA to protein involves a complex array of regulatory factors, including post-transcriptional modifications, RNA shearing, RNA stability, and protein degradation, which can result in discrepancies between RNA and protein levels. For instance, researchers in one study detected stimulator of interferon genes (STING) mRNA in hepatocytes, but did not detect STING proteins [36, 37]. This is particularly relevant in the liver, where endogenous signalling molecules can significantly impact liver immune tolerance, yet may not be revealed by scRNA-seq [38]. Therefore, to gain a comprehensive understanding of the transcriptome and proteome of individual cells, it is crucial to combine scRNA-seq with single-cell protein-based techniques, such as cellular indexing of transcriptomes and epitopes by sequencing (CITE-seq), cytometry by time-of-flight detection (CyTOF), and genetically encoded fluorescent probes.

**Fig. 3:**
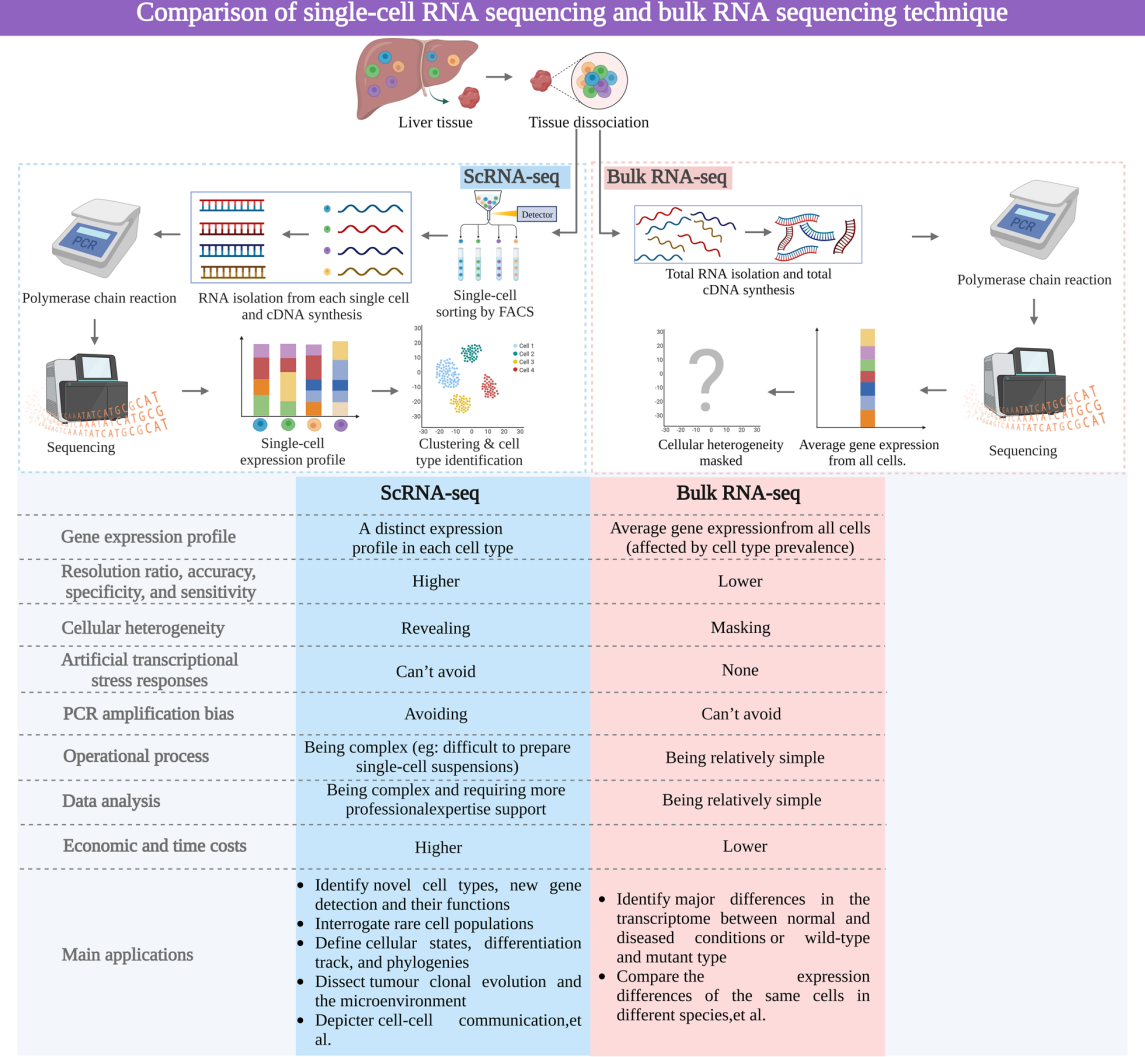
Comparison of scRNA-seq and bulk RNA-seq in the liver. *FACS* fluorescence-activated cell sorting

Thirdly, compared with bulk sequencing, scRNA-seq is far more expensive. Although the experimental costs have been reduced to some extent due to the occurrence of high-throughput scRNA-seq workflows, it is still an expensive technique, costing up to nearly $3000 per sample. Lastly, data analysis of scRNA-seq is time-consuming and requires professional expertise. The specialised knowledge required to conduct these bioinformatic analyses is not trivial. However, standardisation of single-cell analyses and the establishment of sequencing and analysis cores at major research institutes enable clinical biologists to bypass these obstacles step-by-step. Thus, when designing single-cell sequencing experiments and workflows, several essential factors must be considered to obtain the most informative and credible data from each experiment. The advantages and disadvantages of scRNA-seq and bulk sequencing are compared in Fig. 3.

Moreover, the liver’s distinctive characteristics pose methodological challenges and impact scRNA-seq outcomes. Hepatocytes have an abundance of various enzymes, particularly RNA enzymes, which can degrade RNA and influence downstream analysis. Traditional dissociation methods usually employ mixed enzymes at 37 °C, a temperature at which the liver enzymes are highly active, which leads to transcriptional bias. However, the use of cold protease to dissociate tissues at low temperatures has been demonstrated to enhance cellular activity and significantly reduce gene artefact expression [33]. To further preserve RNA integrity, it is viable to add nuclease inhibitors, such as SUPERaseIn and EDTA, to the lysis buffer [39, 40]. Additionally, under high-fat conditions, the gene expression of liver cells may be significantly altered, resulting in the up- or downregulation of certain genes related to fat metabolism [41, 42]. This process may obscure signals from other cell types, thus introducing bias into single-cell transcriptome sequencing results. The use of degreasing reagents, such as Triton X-100, in the cell lysis process can effectively dissolve cells and remove lipids to ensure the acquisition of high-quality RNA [39, 43]. Lastly, hepatocytes are particularly rich in mitochondria, leading to elevated levels of mitochondrial RNA that may interfere with the identification of other RNAs. To address this issue, RNA sequencing data analysis can incorporate unique molecular identifier (UMI) technology to eliminate multi-copy RNA [44]. In essence, single-cell transcriptome technology encounters distinct obstacles when detecting liver tissue, necessitating meticulous procedures for sample collection, processing, separation, screening, and implementing specialised techniques to manage the liver’s high lipid and enzymatic properties, thereby ensuring the precision and dependability of sequencing outcomes.

### From liver to single cell

#### Liver dissociation method

**Fig. 4:**
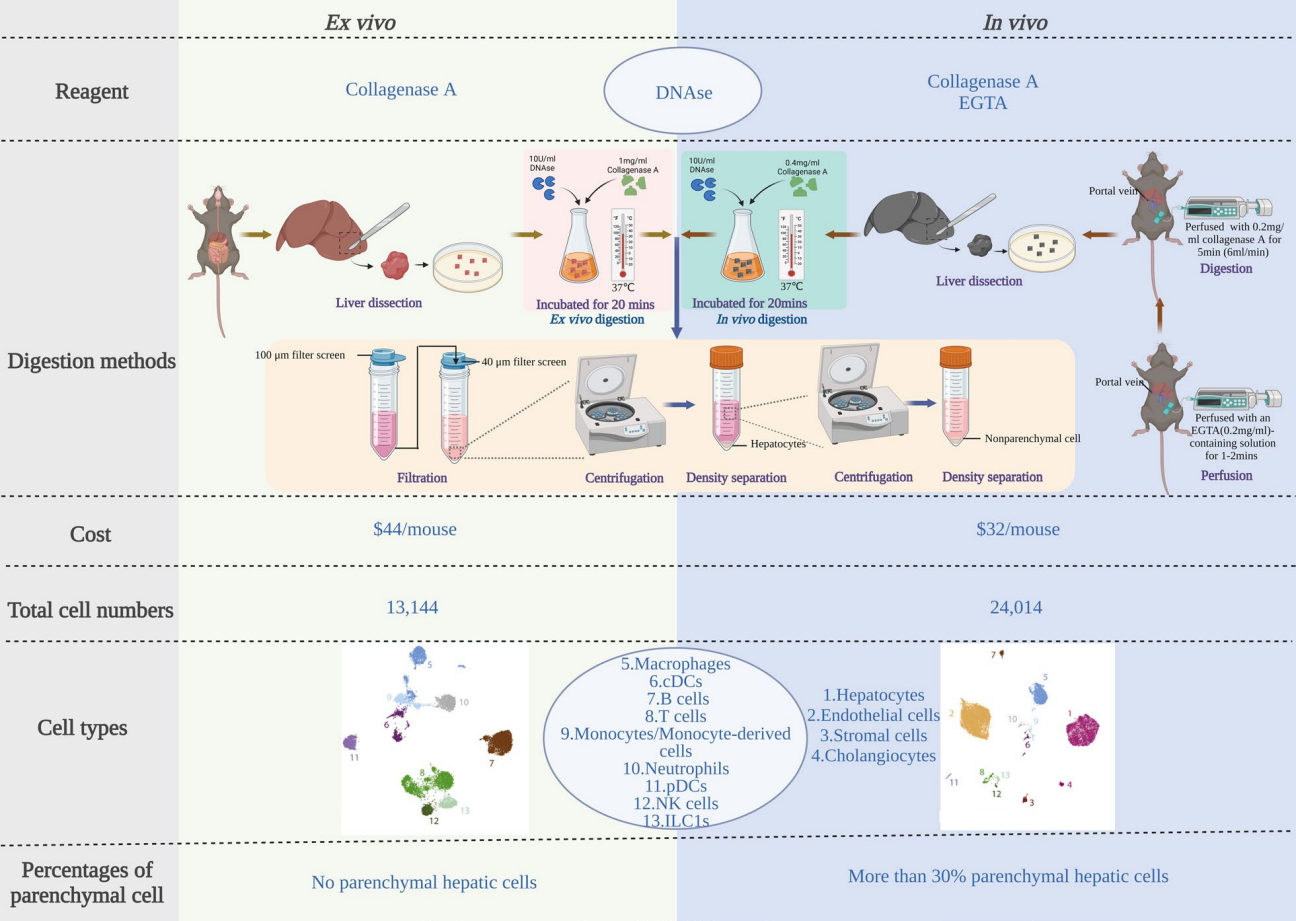
Comparison of two different methods of liver dissociation: ex vivo and in vivo. This table is summarised from REF [41]. *DNAse* deoxyribonuclease, *EGTA* ethylene glycol tetraacetic acid, *cDCs* conventional dendritic cells, *pDCs* plasmacytoid dendritic cells, *NK cells* natural killer cells, *ILC1s* type 1 innate lymphoid cells

For a scRNA-seq experiment, a high quality, viable cell suspension of the liver needs to be obtained through tissue dissociation and cell isolation. Liver dissociation is challenging, and the conventional dissociation method usually causes low hepatic cell vitality and alterations in the genuine ratio and cell types. The basic structural units of the liver are complex in composition, with vigorous metabolism, and cell vitality rapidly decreases after isolation. To address this problem, a study examined the optimal method for retrieving the maximum possible number of high quality hepatic cells [41]. Hepatic cells were isolated via ex vivo or in vivo enzymatic digestion using murine livers. Briefly, for ex vivo digestion, mouse livers were isolated, cut into small pieces, and incubated with deoxyribonuclease (DNAse) and collagenase A with gentle shaking (Fig. 4). For in vivo digestion, the livers were first perfused with an ethylene glycol tetraacetic acid (EGTA)-containing solution after retrograde cannulation, followed by perfusion with collagenase A. The liver was removed from the body, minced, and incubated with a combination of DNAse and collagenase A. Regarding the number of genes/cells, no differences were observed between the two digestion methods. However, there was a remarkable difference in the total cell numbers, cell types, and the cell percentages between the two digestion methods (Fig. 4). Notably, there is still an obvious discrepancy in the number and percentage of cells obtained from the digestion methods compared to that in the genuine liver [18, 45]. For example, parenchymal cells such as cholangiocytes which make up 3–5% of all liver cells [46] consist of only 199 (0.64%) of 8444 cells from the scRNA-seq map [41]. Thus, an optimal and unified method for retrieving all hepatic cells approximated to the genuine state is urgently required to start the scRNA-seq process and reduce bias in different research groups.

#### Single-cell capture strategy

Before studying a single cell, we must first capture it reliably [47]. Currently, several vendors have provided technologies for single-cell capture. Early stage strategies include limiting dilution [48], fluorescence-activated cell sorting (FACS) [49], micromanipulation using an inverted microscope, and the use of motorised micromanipulation or laser microdissection [50]. Although every method has its advantages, their shortcomings are also evident, including the complex procedures, high capture cost, lack of suitability for micro samples, and susceptibility to errors during calculations [51]. In recent years, single-cell sequencing has entered has considerably progressed. There are two strategies for achieving high-throughput single-cell capture. One is microfluidic chip technology, represented by 10X Genomics [52], Drop seq [53] and inDrop [53]. The other is microwell technology, represented by BD Rhapsody [54] and Seq Well [55] (Fig. 2B). High-throughput microdroplet-based microfluidic technologies are used to capture single cells in oil droplets through microfluidic chips, e.g., 10X chromium. The core idea is to assign different barcode sequences to different cells [52]. When building a library, nucleic acid molecules with the same barcode sequence are considered to be from the same cell; therefore, a library can be constructed for hundreds of cells at a time while distinguishing them smoothly [52]. Microwell plate technology involves depositing a single cell and magnetic bead into a micropore. The sequence structure of the magnetic bead is similar to that of 10X Genomics, and the poly A tail of free Mrna can be captured [56]. The advantages of the two methods are high cell flux, fast cycling, low cost, high cell capture efficiency, and simple operation of commercial instruments [56]. In summary, the selection of a single-cell capture strategy largely relies on the cell type of interest, its prevalence in the tissue or organ, and costs.

### scRNA-seq data analysis

Bioinformational analysis of data is the central process for expanding the application scope of scRNA-seq in the medical sciences. The pipeline for scRNA-seq data analysis primarily includes four consecutive analysis steps: data pre-processing, general analysis, exploratory analysis, and optional analysis [57]. According to the data source and sequencing platform, the basic formats of raw sequencing data for scRNA-seq are the FASTQ or BCL formats [58]. The pre-processing of raw data includes quality control, alignment, and UMI (transcript) qualification [44]. After data preparation, a general analysis is conducted, including low-quality cell elimination, normalisation, highly variable genes (HVGs) selection, batch rectification, dimension reduction, clustering, and cell type annotation [16]. Furthermore, various exploratory analyses are conducted based on the research purpose, including differential expression analysis, functional enrichment analyses such as gene ontology (GO) and Kyoto Encyclopedia of Genes and Genomes (KEGG) enrichment, gene set variation analysis (GSVA), transcription factor prediction, and cell trajectory, cell–cell communication, and cell cycle analyses [59]. In addition to exploratory analyses, many other significant issues require more attention and in-depth exploration. For instance, multidimensional analysis can be conducted by combining scRNA-seq and clustered regularly interspaced short palindromic repeats (CRISPR) screening [60]. Integrated analysis of scRNA-seq and multiomics can also be applied, including single-cell whole-genome sequencing [61], single-cell assay for transposase-accessible chromatin using sequencing (scATAC) [62], single-cell methylome and transcriptome sequencing (scMT) [63, 64], cellular indexing of transcriptomes and epitopes by sequencing [65, 66], single-microbe genomics [67], and spatial transcriptomics [68]. Application of conjoint analysis of these aforementioned techniques allows a better and deeper understanding of core biological and pathological processes and mechanisms, which opens a new avenue for the future development of single-cell technology. The major analysis workflow for scRNA-seq data is summarised in Fig. 2E. More details regarding each module can be found in other reviews [16].

### Single nucleus RNA sequencing (snRNA-seq) in liver analysis

The limitations of scRNA-seq include difficulty in tissue preservation and dissociation to obtain single-cell suspensions, inapplicability to sample types containing large cells, applicability to fresh tissue samples only, and artificial stress responses [69]. Recently, researchers have focused on snRNA-seq in liver research [18]. Notably, snRNA-seq can avoid the cell dissociation step by using detergents to directly release nuclei from intact cells [18]. Instead of sequencing all the Mrna in the cytoplasm, snRNA-seq captures only the transcripts in the nucleus [70]. Thus, it is understandable that these differences affect the sensitivity of these methods in delineating the subtypes of the respective cell types. ScRNA-seq and snRNA-seq have been compared while conducting in liver analysis [41]. Typically, a lower number of genes/cells was yielded by snRNA-seq than by scRNA-seq. However, this did not prevent the identification of distinct cell types, as both scRNA-seq and snRNA-seq identified highly expressed genes in each population [41]. Nevertheless, gene expression was often higher in scRNA-seq than in snRNA-seq. Additionally, an expression pattern of digestion-associated and snRNA-seq-associated genes was observed across different cell types [41]. Despite the fact that snRNA-seq best recapitulated the cell frequencies observed in vivo, it was inferior to scRNA-seq in terms of genes/cell. In liver analysis, snRNA-seq might have advantages in detecting a greater number of cholangiocytes, hepatocytes, and mesenchymal cells using frozen liver tissues [19, 71]. Notably, snRNA-seq only captures Mrna in the nucleus, and hence, might lose important information on biological processes involved in RNA stability, Mrna processing, and metabolism. In addition, a comprehensive atlas of the intrahepatic immune landscape is essential for interpreting liver pathogenesis. However, plenty of important information regarding immune cells was lost from the snRNA-seq data. For example, none of the T cell or B-cell receptor components were detected in liver samples using snRNA-seq [18]. Therefore, it is recommended that studies investigate intrahepatic immune cell populations using scRNA-seq. As each approach has its strengths and weaknesses, the optimal method depends on the scientific questions being addressed.

### Spatial transcriptomic techniques used in liver analysis

ScRNA-seq entails the dissociation of organs and tissues into single-cell suspensions, resulting in the complete loss of spatial information and the additional risk of undesirable transcriptional changes [32, 72]. The liver is a highly complex organ with distinct spatial properties. A deep understanding of spatial heterogeneity is vital for comprehending hepatic structure and function, both in homeostasis and disease. Previous studies have usually performed fluorescence in situ hybridisation [73], immunohistochemical staining, selective perfusion isolation, or laser capture microdissection to define unique transcriptional and protein patterns of perivenular and periportal hepatocytes in different regions of the liver using zonal “landmark genes” [74]. Owing to the limitations of methods available in the early stage, novel approaches are generalising the technique of spatial transcriptomics (ST) to eliminate dependency on landmark genes or nearby cells [75, 76] (Table 1). ST has unique advantages in obtaining high-resolution landscapes of spatial Mrna expression patterns across tissue sections and overcoming deficiencies related to tissue dissociation. Therefore, combining ST data from liver sections in their bona fide tissue location with previously recognised knowledge of liver zonation [77, 78] allows the spatial annotation of structures containing small mixtures of cells in the liver microenvironment (lobule) and liver macroenvironment (tissue). Additionally, ST application across whole liver sections can help identify novel structures that may play vital roles in the overall architecture of the liver, and which may be lost when using protocols that do not allow analysis of the structures in the spatial context.

**Table 1:** Application of high-throughput single-cell and spatial technologies in the liver

Technologies	Molecular layer	Application	Ref.
ScRNA-seq	Transcriptomic	Applicable for fresh tissue samples only and artificial transcriptional stress responses	[69]
SnRNA-seq	Transcriptomic	Application to fresh and frozen samples, particularly those that are difficult to dissociate into single-cell suspensions. Can provide data on difficult to isolate cells with some loss of transcriptional depth and the cytoplasmic RNA fraction	[16]
Spatial transcriptomics	Transcriptomic	Used to eliminate dependency on landmark genes and adherent cells, and provide spatial annotation of structures	[75, 76]
Sc ATAC-seq	Epigenetic	Unbiased detection of epigenetic landscape across the human genome. Capture of early lineage-determining epigenetic features may allow for a higher resolution when identifying cell subsets than with transcriptomic data	[62]
Single-cell immune profiling	Transcriptomic and TCR sequencing	Enables annotation of invariant T-cells, tracking the expansion of T- and B-cells and the linking of antigen receptor sequences to lymphocyte transcriptome	[190]
Sc MT-seq	Multiomic: epigenetic and transcriptomic	Used to detect transcriptome, methylome, and single nucleotide polymorphism information within single cells to dissect the mechanisms of epigenetic gene regulation	[63, 64]
Sc CITE-seq	Multiomic: transcriptomic and proteomic	Used to provide detailed characterization of cellular phenotypes and be readily scale as the throughput of single-cell sequencing approaches increase	[65]
Single-microbe genomics	Genomic	Contribute high-throughput culture-free capabilities to investigate genomic blueprints of complex microbial communities with single-microbe resolution	[67]

*ScRNA-seq* single-cell RNA sequencing, *ScATAC-seq* single-cell assay for transposase-accessible chromatin using sequencing, *ScMT-seq* single-cell methylome and transcriptome sequencing, *snRNA-seq* single nuclear sequencing, *ScCITE-seq* single-cell cellular indexing of transcriptomes and epitopes by sequencing, *TCR* T cell receptor

### General workflow for integrated analysis of scRNA-seq and ST data in the liver

The integrated analysis of single-cell techniques and ST can provide a deeper understanding of the function and interaction of liver cells in both physiological and pathological states, and are reviewed in detail in the following sections. The concise workflows combining scRNA-seq and ST techniques typically begin with establishing cell subtypes through dimensionality reduction and clustering of scRNA-seq data. Cell subpopulations are then localised through deconvolution and mapping [79, 80]. Spatial barcoding data are usually subjected to deconvolution, while single-cell resolution spatial data (such as high-plex RNA imaging (HPRI) data) are typically subjected to mapping to localise scRNA-seq subpopulations. Next, algorithms that evaluate the spatial arrangement of localised subpopulations further assess the ligand–receptor interactions predicted from scRNA-seq data. Given that much cellular crosstalk, notably juxtracrine and paracrine communication, is spatially restricted, ST data are well suited to evaluate the reliability of the ligand–receptor interactions computed from scRNA-seq [81, 82]. Finally, the integrated results can be visualised through the creation of heat maps, scatter plots, and two-dimensional graphs, among other methods. For more information on each module, please refer to a recent review article published in “Nature Reviews Genetics”, which explores the attempts to integrate scRNA-seq with ST techniques, including the use of emerging integrated computational methods, and proposes effective ways to combine current methods [83].

## Single cell and ST in a normal liver

The human liver is the largest organ of the body. It is made up of parenchymal cells, such as hepatocytes and cholangiocytes, that constitute 80% of the liver mass [84], and nonparenchymal cells, such as HSCs, LSECs, and recruited and tissue-resident immune cells [85]. ScRNA-seq enables the simultaneous detection of parenchymal and nonparenchymal cells to generate an atlas in the normal liver, provides novel insights into the phenotypic zonation of hepatocytes, and improves our understanding of rare cell types, such as LSECs [73], cholangiocytes [73], and hepatic progenitor cells [86] (Fig. 1B). Additionally, the integration of scRNA-seq and ST can generate high-resolution maps of cell subpopulations in tissues. This combination can help us better understand the changes in cell types and gene expression in different regions of the liver.

### A single-cell perspective of normal liver cell atlas

An important application of scRNA-seq is to generate a full-scale perspective of a normal liver cell atlas. Since 2018, several independent studies have performed single-cell analysis in healthy human livers at high-throughput resolution [15, 41, 59, 72, 73, 87–90]. MacParland et al. were the first to publish a study on the normal human liver utilising scRNA-seq technology [73]. Liver grafts were obtained from five neurologically deceased donors with systemic inflammation from brain death but normal liver histology, and 8444 liver cells were profiled. Twenty different cell populations were characterised, including hepatocytes, endothelial cells, cholangiocytes, HSCs, B cells, conventional and non-conventional T cells, and two distinct populations of resident macrophages [73]. Liver samples used to generate a normal liver cell atlas have also been acquired from individuals of both sexes across a wide range of age groups (from 21 to 65-years-old), and from those with diverse underlying medical conditions [72, 88, 90]

It is extremely difficult to obtain fresh, healthy human liver tissue of good quality in the field. Therefore, a recent study has compiled, integrated, and analysed available scRNA-seq data from 28 healthy human liver samples. Moreover, they have established an online cell browser that provides easy and open access to transcriptional data of a diverse range of annotated parenchymal and nonparenchymal cells [91]. The combined database includes 26 clusters of 36,188 human hepatic cells [91]. Notably, an R Shiny approach has been developed for interactive visualisation, and scientists can access the website (http://liveratlas-vilarinholab.med.yale.edu). This innovative approach offers a user-friendly experience and interactive visualisation of gene expression for each cell (sub)type, ranging from abundant to rare liver cell subpopulations. Furthermore, this pioneering web tool furnishes detailed resources on which and what proportion of cell (sub)types express a gene of interest.

In summary, using novel single-cell technologies, these studies provide new insights into the transcriptomic structure of the human liver in a physiological state, revealing previously unattainable information that is highly valuable for the liver research community worldwide. However, there are still many aspects of liver biology that have barely been studied and several problems that need to be addressed in the future. First, the normal livers are obtained from patients with a variety of underlying medical conditions, in other words, they are not really “normal”. Additionally, the dissociation and handling of liver tissue are thought to induce changes in gene expression in liver cells. Finally, the dynamics of the normal liver atlas due to gender and age need to be elucidated more thoroughly.

### Landscape of intercellular crosstalk

ScRNA-seq has provided many innovative insights into cellular heterogeneity and function in liver physiology. However, pathological processes such as ischaemia, inflammation, anti-infection, fibrosis, regeneration, autoimmunity, and tumourigenesis are complicated, with a highly organised interactome between specific subgroups of multiple cell types that are likely to play crucial roles in disease progression. Therefore, to discover new and effective therapeutic targets, it is crucial to understand the complex interactions present in different liver diseases. Importantly, scRNA-seq data not only define the characterisation of different cell types but also provide information on which cell subgroups express specific genes of interest and to what extent. Accordingly, we can further analyse scRNA-seq data to study the underlying ligand–receptor interactions between distinct cell types, model the interactome, and highlight cellular and molecular mechanisms that might drive disease progression.

A number of bioinformatic methods have emerged to carry out interactome analysis on scRNA-seq data [92–94]. Unbiased CellPhone DB analysis [93] is an approach that infers inter- and para-cellular crosstalk from the combined expression of multi-subunit ligand–receptor complexes between cell clusters. To clarify the molecular mechanisms of an immune checkpoint inhibitor (ICIs)-related hepatitis, CellPhone DB analysis was carried out to identify the ligand–receptor communications between T cells and myeloids in the liver of an ICI-induced hepatitis murine model [95]. Significant ligand/receptor pairs with T cell activation cytokines TGFB, IL21, IL18, and IL15 were found between T cells and myeloids and between T cell subgroups [95]. The CellPhone DB algorithm has also been used to investigate the intra-scar activity of several pro-fibrogenic signalling pathways between endothelial cells, collagen-producing mesenchymal cells, and scar-associated macrophages in human fibrotic livers [90]. Notably, during fibrosis, high levels of the Notch ligands Delta-like ligand 4 (DLL4), Jagged 1 (JAG1), and Jagged 2 (JAG2) expressed by endothelial cells can signal through Notch receptors expressed by mesenchymal cells [90]. Single-cell secretome gene analysis was performed on hepatocytes and nonparenchymal cells isolated from the livers of an NAFLD mouse model [42]. By combining scRNA-seq data with a ligand–receptor interactome database [96], a highly correlated signalling network among major hepatic cell types was generated, indicating potential paracrine and autocrine connectivity with a high speciality in the mammalian liver [42].

In summary, compared with traditional bulk RNA sequencing, scRNA-seq data analysis provides a sensitive approach for delineating precise and complicated cellular sources of ligands and receptors and elucidating the roadmap of intercellular communication within the mammalian liver. Future challenges include functional assessments of the predicted signalling pathways and the precise elucidation of their biological functions in health and disease.

### Liver zonation and function based on a combination of spatial and single-cell transcriptomes

The hepatic lobules, a fundamental functional unit in the mammalian liver, take on a hexagonal shape and contain hepatocytes arranged in plates along the radial lobule axis, with the central and portal veins situated at either end [97, 98]. In accordance with this spatial variation, versatile functions of the liver vary non-uniformly inside the hepatic lobules along this porto-central axis, a phenomenon termed “zonation” [84, 99]. Therefore, according to different hepatic functions and anatomical locations, the hepatic lobule is classically divided into three different zones [75]. Zone 1 surrounds the portal tract where oxygenated blood from the hepatic arteries enters. Zone 3 encircles the central veins, which is hypoxic but receives a nutrient-rich blood supply from the gastrointestinal tract. Zone 2 is located between Zone 1 and Zone 3 (Fig. 1C).

Our knowledge of hepatic cell organisation within these zones was deepened by spatially resolved transcriptomic analysis using scRNA-seq. By combining scRNA-seq with single-molecule RNA fluorescence in situ hybridisation (smRNA-FISH), Halpern et al. performed spatially resolved RNA sequencing to study liver zonation in mice [100]. This study indicated that liver zonation was mainly determined by the oxygen gradient and classical WNT signalling [101], and also by rat sarcoma protein (RAS) signalling to a lesser extent. Intriguingly, a substantial number of spatially zonated genes were not predicted to be downstream targets of these signalling pathways, suggesting the need for the identification of many other molecular signalling pathways important for liver zonation identification. Notably, the mouse interzonal hepatocyte marker genes identified are not applicable to human liver data [18, 73].

Building on the previous study [100], the concept of ST to dispel dependency on landmark genes or adherent cells was generalised by updated approaches. One of the current strategies is to fix MrnA molecules within the cell and introduce primers with barcode sequences, followed by rolling circle amplification (RCA) to increase the local copy number [102, 103]. Although a single-cell resolution is yet to be achieved, as recently reported by Sun et al., spatial transcriptomic techniques enable unbiased reconstruction of metabolic liver zonation [104]. Moreover, to highlight the spatiotemporal metabolic organisation of the liver, ST and scRNA-seq were performed in combination on individual liver cells obtained via perfusion from 10 ad libitum fed mice across the diurnal circadian cycle at different time points [22]. ST was also employed to resolve the spatial correlations of vascular parts involved in liver zonation and to explore previously unidentified structures in the mouse liver [20]. Tracking the expression of transcriptional markers associated with zonation along the lobular axis computationally enables the study of zonation gradients in physical positions and allows the prediction of vein-type identity according to the expression profiles of neighbouring spots. In addition, the presence of transcriptionally distinct structures, cluster 5, was discovered in liver tissues, consisting of a small number of spots with distinct spatial localisation, which express mesenchymal cell-marker genes such as *Vim* and *Gsn* [105]*.* This cluster has not been identified in previous transcriptomic studies, mainly owing to the cell rarity contributing to these structures [20].

In addition to MrnA, other cellular features in hepatic cells may have similar spatial heterogeneity, including proteins, metabolites, and regulatory molecules such as microRNAs (miRNAs). Understanding miRNA and protein space distribution are essential to deciphering the contributions of hepatic cells to liver development, metabolism, regeneration, fibrosis, infection, and cancer [106]. Itzkovitz et al. adopted “spatial sorting”, a generic method, to illustrate the zonation of hepatocyte proteins and miRNAs [104]. Their study provides a generalised spatial atlas of protein and miRNA zonation, identifying core hepatocyte-donated miRNAs, such as miR-30a-5p and miR-122-5p [104]. Through a combination of miRNA and target MrnA levels, they also identified potential regulatory interactions that could mediate the degradation of zonated MrnA. This provides an important resource for future studies because miRNAs are highly dynamic and zoned during disease progression of the liver, such as fibrosis, viral infection, and cancer [107–109]. Moreover, spatial identification of metabolic enzymes and signalling pathways using proteomics and in silico techniques will definitely increase our knowledge of hepatotoxicity and can greatly improve the prediction accuracy of drug absorption, distribution, metabolism, and excretion [104, 110].

Therefore, ST approaches have profoundly improved our understanding of the functional specialisation of human livers and could be used to establish how this division of labour may be restored after disruptions caused by liver injury [72, 73]. For further anticipation, ST will greatly benefit studies addressing liver development, sexual dimorphisms of liver zonation, immunity, and general pathology in mammals, particularly humans.

## Spatial and single-cell transcriptomics in the liver during infection

The liver is the largest immune organ of the human body. Because of acute or chronic infection, liver inflammation results in necrotic hepatocyte death [111]. Persistent hepatic damage leads to progressive fibrosis, disrupted liver architecture, cirrhosis, and subsequent tumorigenesis. The identification of single-cell processes throughout the course of infection is hindered by restricted access to human liver tissue longitudinally over time and, until recently, limited options for investigating rare and potentially disease-driving cell populations. However, high-throughput spatial and single-cell technology has shed new light on the mechanisms underlying liver function during infection.

### Hepatitis B virus (HBV) infection

**Fig. 5:**
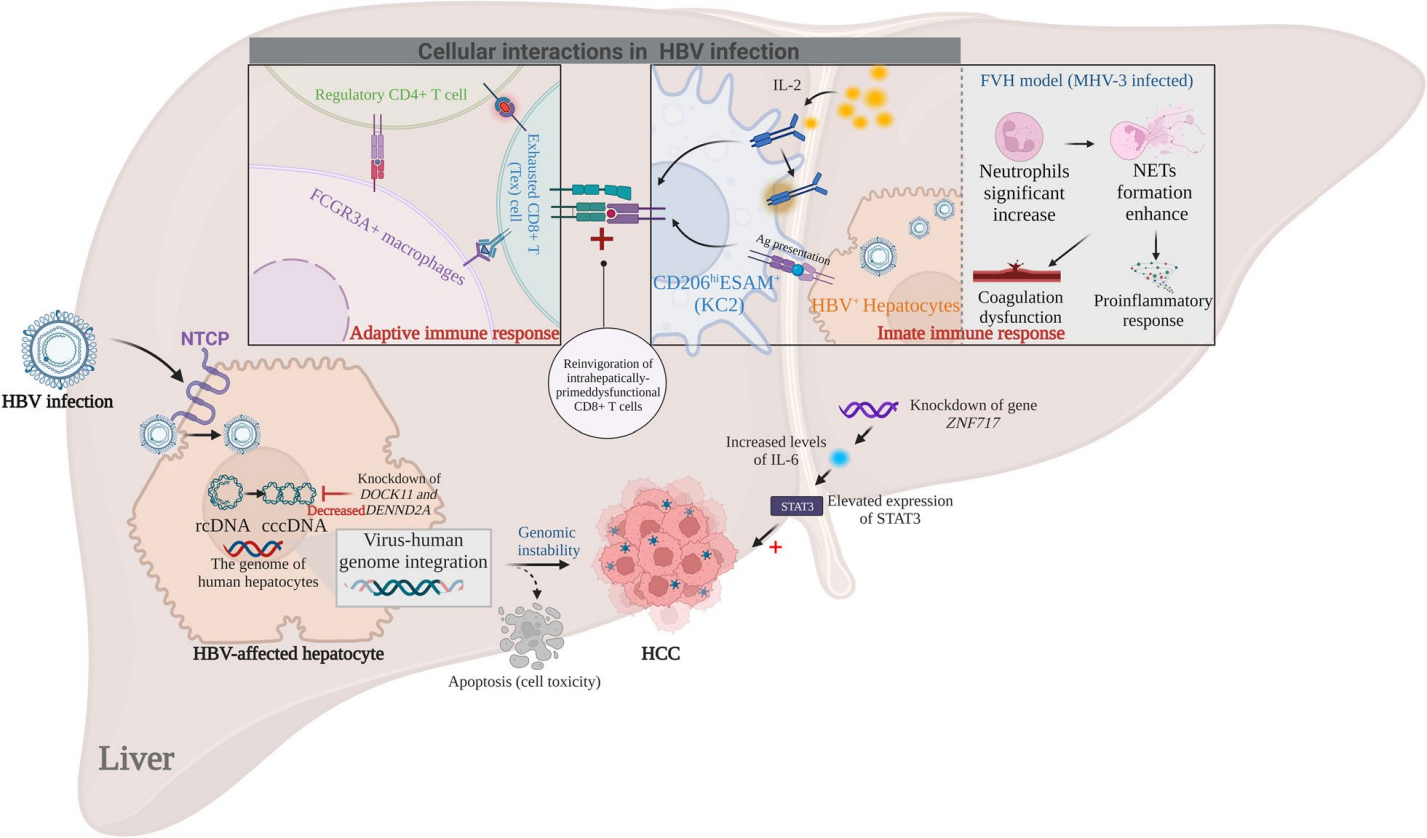
Underlying mechanism in HBV infection using ScRNA-seq. *cccDNA* covalently closed circular DNA, *FVH* fulminant viral hepatitis, *HCC* hepatocellular carcinoma, *HBV* hepatitis B virus, *IL-2* interleukin-2, *KC* Kupffer cell, *MHV-3* murine hepatitis virus strain-3, *NTCP* Na^+^-taurocholate cotransporting polypeptide, *NETs* neutrophil extracellular traps, *rcDNA* relaxed circular DNA

HBV infection is a serious health and economic burden worldwide [112]. Currently, there is no radical cure for HBV infection because the virus remains in a latent state as stably inactivated covalently closed circular DNA (cccDNA) within host cells [113]. Therefore, shedding novel insights into the underlying mechanism for the maintenance of HBV cccDNA is of great significance in identifying new paths of HBV replication (Fig. 5). Using the scRNA-seq analysis, Hashimoto et al. characterised *DOCK11* and *DENND2A* as key genes linked to the preservation of HBV cccDNA in 2325 primary human hepatocytes infected with HBV [21]. The amounts of HBV DNA and cccDNA both decreased below the limit of detection under the depleted expression of these two genes, which revealed an important role of these genes in HBV maintenance in human hepatocytes [21] (Additional file 1: Fig. S1A). However, the underlying mechanism remains poorly understood and needs further exploration. Furthermore, to obtain an unbiased and full-scale landscape of intrahepatic immunological characteristics and associations with disease status in HBV-infected patients, scRNA-seq was performed in the liver and blood during different stages of hepatitis B infection, including immune tolerance, immune activity, acute recovery, chronic resolved, and healthy controls [114] (Additional file 1: Fig. S1B). Diverse immune cell subgroups with different sources and cellular interactions have been observed in different disease statuses in HBV infection. This study highlighted the communication between exhausted CD8+ T (Tex) cells, regulatory CD4+ T cells, and FCGR3A+ macrophages. This communication contributes to immune failure during HBV maintenance through mediating HLA class I molecules together with their receptors such as leukocyte immunoglobulin-like receptor, and may be useful in guiding the progress of immunotherapy (Additional file 1: Fig. S1B). In addition to the adaptive immune response, the innate immune cells play a significant role in HBV infection. To delineate the function of KCs in HBV infection, live CD45+CD64+F4/80+ liver macrophages were sorted by flow cytometry from C57BL/6 mice and subjected to scRNA-seq analysis using the Smart-seq2 pipeline [23]. Two distinct clusters of KCs among liver-resident macrophages were identified, and the CD45+F4/80+CD11bint TIM-4+CD206+ESAM+ cells were poised to respond to IL-2 treatment and cross-present viral antigens contained within circulating virions or hepatocytes. Therefore, specific subgroups of KCs were identified as regulating the signalling pathways that lead to the hepatocellular priming of HBV-specific CD8+ T cells, which acquired pathogenic effector and antiviral functions following exogenous IL-2 treatment (Additional file 1: Fig. S1C).

Indeed, scRNA-seq can provide abundant transcriptomic data to study molecular events and cell heterogeneity at the single-cell level. However, due to hereditary deficiency, high-expression genes in the library are sequenced more frequently, and rare genes can be easily ignored. Therefore, it is challenging to acquire transcript information from low-abundance or rare genes in the presence of highly abundant genes [115]. Tyrrell et al. developed a CRISPR-CRISPR-associated nuclease 9 (CRISPR-Cas9) assay to deplete high-abundance transcripts, resulting in preferential enrichment of low-abundance genes, such as HBV genes in human hepatocytes. By incorporating CRISPR-Cas9 with scRNA-seq in hepatocytes infected with HBV, the three most abundant transcripts were successfully depleted, which enabled selective enrichment of the HBV transcript and subsequent HBV RNA sequencing in more than 74% of the cells, compared to the detection of HBV RNA in only 0.6% of the cells through direct sequencing without CRISPR-mediated enrichment [116] (Additional file 1: Fig. S1D). The technical improvement further enabled the study of HBV infection and interferon treatment of the HBV-infected Huh7.5-NTCP cell model.

The single-cell assay for transposase-accessible chromatin using sequencing (ScATAC-seq) is a novel technique that detects open regions and identifies regulatory regions of chromatin at the single-cell level, making it a broad region of single-cell high-throughput technologies [117]. The corresponding transcription factors or other regulatory proteins bind to open regions of chromatin and directly affect intracellular gene expression [118]. To better understand the HBV-associated cell types, different open regions of chromatin, and specific gene expression regulatory networks in patients with chronic hepatitis B (CHB), Dai et al. performed ScATAC-seq on 8016 peripheral blood mononuclear cells (PBMCs) derived from normal control (NC) individuals and CHB patients [119]. Specific leukocytic subgroups associated with CHB were found to be located in B-0 and T-3 clusters. Additionally, the potential mechanisms of the transcription factor (TF) motifs IRF2 and FOXC2, which are associated with B-0 and T-3, respectively, were elucidated in the occurrence of CHB (Additional file 1: Fig. S1D). Therefore, a more systematic and distinctive gene regulatory network was constructed by combining scRNA-seq and ScATAC-seq [119].

HBV can also cause fulminant viral hepatitis (FVH), a deadly disease that lacks an effective treatment, and its pathogenesis is not fully understood [120]. Immune response plays a crucial role in the pathological progression of liver failure, yet the entire immune microenvironment landscape of infected livers is important for effective strategy discovery [121]. Wang et al. constructed an scRNA-seq study of a mouse model of FVH due to murine hepatitis virus strain-3 (MHV-3) infection. ScRNA-seq revealed that neutrophils were the only innate immune cells exhibiting a significant increase in the liver in response to MHV-3 infection when the mice had severe systemic inflammation. Neutrophils extracellular traps (NETs) exacerbated liver damage by promoting fibrin deposition and inflammation, which were regulated by the FGL2-mucolipin 3-autophagy axis (Additional file 1: Fig. S1E). In summary, targeting NETs could offer a novel approach for treating FVH.

The underlying mechanisms of HBV-induced liver damage are multifarious; one of the well-known mechanisms is the HBV genome integration into the genome of human hepatocytes [122, 123]. Virus–human genome integration results in genomic instability and is a high-risk factor for the development of liver tumorigenesis (Fig. 5). In the context of hepatocellular carcinoma (HCC), the heterogeneity of HBV integration has been investigated using single-cell genome sequencing [124]. HBV integration was remarkably consistent in all cells of a single-nodular HCC with portal vein tumour thrombosis, supporting the notion that HBV integration is an early initiating event in hepatotumorigenesis. Moreover, a potential driver gene *ZNF717* was specifically identified in HCC. This gene exhibits a noteworthy mutation rate at both the single-cell and population levels, and acts as a tumour suppressor by modulating the IL-6/STAT3 pathway [124] (Fig. 5).

### Hepatitis C virus (HCV) infection

Although HCV is the world’s most common blood-borne virus, there is currently no vaccine to protect against it [125]. HCV persistence has been established in most infected patients, suggesting that it successfully evades innate and adaptive immune surveillance at multiple levels. Previous standard treatment depends on interferon (IFN)-based therapy; however, it has many adverse side effects and only 50% patients respond. Since 2015, highly effective direct-acting antiviral (DAA) therapy has revolutionised the treatment of chronic HCV infection, with standard cure rates exceeding 95% [126, 127]. Many studies have shown that, following IFN-free DAA treatment, both innate and adaptive immune homeostasis may be partially restored [127–129]. To clarify the role of DAA-mediated HCV eradication in global T cell immune function, Rosen et al. utilised scRNA-seq to characterise the transcriptome of circulating T cells before, during, and after the DAA-mediated HCV cure [130]. They revealed that rapid normalisation of IFN signalling was achieved in T cells with DAA therapy and was well maintained after therapy termination. Several T cell subtypes, including CD4 central memory and effector cells, CD8 effector memory and TEMRA cells, as well as Hi-interferon stimulated gene (ISG) populations, exhibit the most notable transcriptional alterations following this HCV cure. This study identified previously uncharacterised shifts in innate immune and interferon signalling within T cell subpopulations induced by DAA therapy in chronic HCV infection at different time points, providing an abundant data source for exploring the effects of DAA treatment on bulk T cells.

**Fig. 6:**
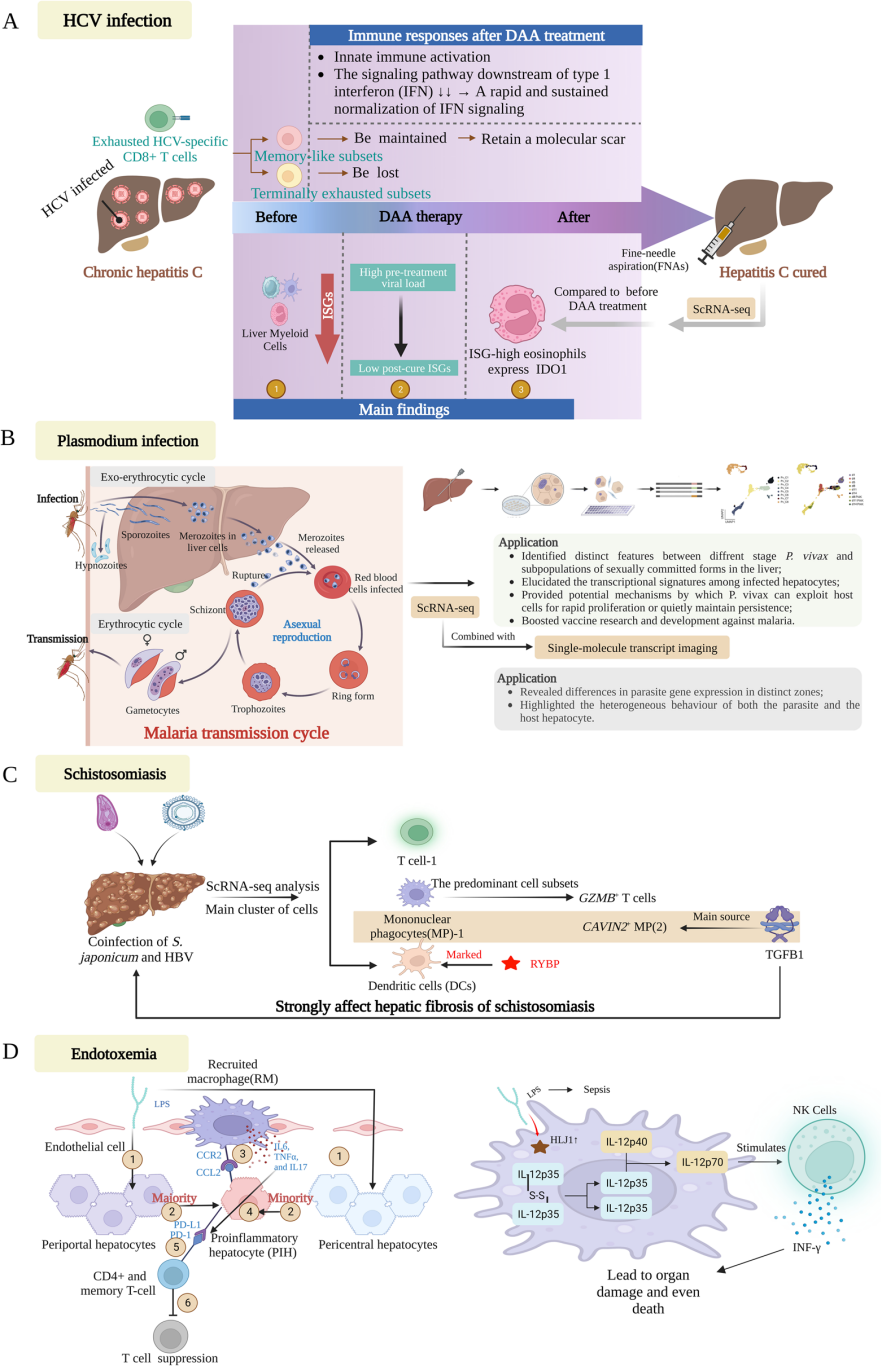
Single-cell perspective of the liver in other infection diseases. *DAA* direct-acting antiviral agents, *DCs* dendritic cells, *HCV* hepatitis C virus, *HLJ1* human liver DnaJ-like protein, *INF-γ* interferon-γ, *LPS* lipopolysaccharide, *MP* mononuclear phagocytes, *MPCC* micropatterned co-cultures, *NKs* natural killer cells, *P. vivax* *Plasmodium vivax*, *PIH* proinflammatory hepatocyte, *YBP* ring1 and YY1 binding protein, *RM* recruited macrophage, *TGFB1* transforming growth factor B1

T-cell exhaustion may serve to limit excessive immunopathology during prolonged antigen stimulation; nevertheless, the potential outcomes of T cell exhaustion may include viral persistence and tumour progression [131–134]. In order to characterise the transcriptional profile, trajectory, and ultimate fate of these exhausted HCV-specific CD8 + T cells during and after the cessation of chronic antigen stimulation in the context of chronic HCV infection, Hofmann conducted scRNA-seq analysis in a well-defined cohort of patients receiving DAA therapy for chronic HCV infection [135]. It demonstrated that memory-like T cells persisted while terminally exhausted T cells diminished after DAA-mediated HCV treatment, leading to memory polarisation of the overall HCV-specific CD8+ T cell response. However, a core signature of exhausted memory-like CD8+ T cells was still detectable, including HCV-specific CD8+ T cells targeting variant epitopes to a lesser extent (Fig. 6A). The aforementioned data delineated a fresh hallmark of T cell exhaustion, which persists as a chronic blemish in CD8+ T cells specific to HCV, even subsequent to the discontinuation of chronic antigenic stimulation. Consequently, in order to elicit complete effector potential, therapeutic targeting of these chronic blemishes, in addition to antigen withdrawal, is imperative.

In addition to adaptive immune response, myeloid cells from liver fine needle aspirates (FNAs) in HCV patients before and after DAA treatment were deeply profiled by scRNA-seq [136]. Subpopulations of liver neutrophils, classical monocytes, non-classical monocytes, macrophages, eosinophils, conventional dendritic cells (cDCs), plasmacytoid dendritic cells (pDCs), and mast cells were comprehensively characterised. Notably, the upregulation of MCM7+STMN1+ proliferating CD1C+cDCs and expected downregulation of ISGs were observed post-cure (Fig. 6A). Programmed cell death-ligand 1 (PD-L1) + ISG-high neutrophils and IDO1-high eosinophils were identified as being crucial for immunoregulatory during chronic infections (Fig. 6A). In addition, three recurring gene programmes, ISG, MHC-II, and S100 were shared by multiple cell types, providing immunotherapeutic insights into HCV infections.

### *Plasmodium* infection

Malaria remains a grave global health concern, afflicting approximately 421 million individuals and resulting in 627,000 fatalities in the year 2020 [137]. *Plasmodium vivax* is responsible for the majority of non-African cases of malaria [137]. There is an obligatory developmental stage of all Plasmodium parasites that infect humans in the liver, where the parasite experiences asexual reproduction known as schizogony within a human hepatocyte before releasing thousands of merozoites into the blood [138]. In the life cycle of *P. vivax* in the liver, some parasites known as hypnozoites give up immediate division in hepatocytes and persist in the liver for days, weeks, months, or even years before initiating schizogony, resulting in a relapse of blood infection [139] (Fig. 6B). Therefore, eliminating *P. vivax* without therapy that directly targets hypnozoite reservoirs is challenging [140]. However, it remains unclear whether the mechanism regulating the formation and dormancy of hypnozoites is the same in different relapsing malarial parasites.

Previously, transcriptome-wide profiling of the Plasmodium during liver stages was performed using traditional bulk RNA sequencing strategies [141]. Although informative, the study only provided insights into average transcriptional expression originating from either mixed (schizont and hypnozoite) or hypnozoite-only populations, instead of any transcriptional variations that might be present between individual parasites. Moreover, the potential impact of the parasite on host cells was not considered, impeding the investigation of host–pathogen interactions. These deficiencies emphasise the need to better understand the differences between individual plasmodia, as well as how the Plasmodium alters hepatic cells. Progress in these fields could facilitate the characterisation of parasite and host factors important for the development of the parasite liver stage.

To comprehensively examine Plasmodium-specific host interactions and potential responses in bystander hepatocytes, Dr. Sangeeta N. Bhatia conducted dual transcriptional profiling of *P. vivax* infection and surveyed host- and state-dependent gene expression patterns in both parasites and hepatocytes [142]. This research utilised a bioengineered human microliver platform to culture patient-derived *P. vivax*, generating a single-cell liver atlas of relapsing human malaria through scRNA-seq analysis. The study identified distinct features of early-, dormant-, mid-, and late-stage *P. vivax*, as well as subpopulations of sexually committed forms in the liver that were previously thought to emerge only during erythrocytic infection [143] (Fig. 6B). *P. vivax* infection inhibits the transcription of functional genes in key hepatocytes and induces an anti-*P. vivax* innate immune response, which includes the dysregulation of IFN and inflammatory signalling pathways. This study provides a foundation for understanding parasite–host interactions and sheds light on the biology of *P. vivax* dormancy and transmission. Moreover, the technology developed in this study can be applied to research other intracellular microorganisms, where low expression of pathogen transcripts or host contamination makes it difficult to perform single-cell studies.

At around the same time, Kyle et al. utilised the scRNA-seq platform of 10X Genomics to characterise the transcriptomic features of both the host and parasite in a *P. vivax* liver-stage model in vitro [144]. Differential gene expression was observed between replicating hypnozoites and schizonts, and previously unobservable variation by bulk sequencing was revealed between individual hypnozoites. Moreover, multiple host genes linked to energy metabolism and antioxidant stress were exclusively upregulated during hypnozoite infection in infected hepatocytes. This study not only elucidated the transcriptional signatures among infected hepatocytes but also provided potential mechanisms by which *P. vivax* can exploit host cells for rapid proliferation or quietly maintain persistence (Fig. 6B), which can inform therapeutic targets against *P. vivax* liver-stage infection.

Previous ex vivo investigations have suggested that the rate of Plasmodium infection may differ among hepatocytes situated in distinct regions (pericentral and periportal) within the liver, owing to spatial heterogeneity of hepatocytes [145, 146]. To identify heterogeneous host and parasite responses, scRNA-seq and single-molecule transcript imaging were combined to characterise the host and parasite temporal expression programmes in a zonally controlled manner for the rodent malaria parasite *Plasmodium berghei* ANKA [147]. The study revealed differences in parasite gene expression in distinct zones, including potentially co-adaptive programmes related to iron and fatty acid metabolism (Fig. 6B). Parasites were found to develop more rapidly in the pericentral lobule zones, and a subpopulation of ‘abortive hepatocytes’ were identified periportally; these appeared predominantly with high parasite inoculum, upregulated immune recruitment, and key signalling programmes. This study provides an ideal application of combined spatial single-cell analysis for understanding the liver stage of Plasmodium infection at high spatial resolution and highlights the heterogeneous behaviour of both the parasite and the host hepatocyte [148].

Single-cell technology has also boosted vaccine research and development against malaria. Dr. Alexandra J. Spencer and her colleagues conducted an in-depth analysis of immune phenotyping and scRNA-seq on the kinetics of peripheral blood samples and thin needle puncture of liver tissue to investigate CD8+ tissue-resident memory (TRM) cells and their circulating counterparts in volunteers who received a novel malaria vaccine treatment called prime-target vaccination [149]. By exploring the heterogeneity among liver CD8+ TRM cells at the single-cell level, two main subpopulations were identified, each sharing expression profiles with blood T cells. This study uncovered the potential of liver TRM-like cells as a protected object by liver-stage malaria vaccines, especially those adopting a prime-target approach. Phase III clinical trials of liver-stage malaria vaccines are currently underway, and a reproducible correlation of protection would be particularly valuable (Fig. 6B).

### Schistosomiasis

Schistosomiasis, whose pathogenesis remains unclear, is a widespread helminth disease that causes acute and chronic injury and poses a heavy social and economic burden on people worldwide [150–152]. Advanced schistosomiasis often progresses to liver fibrosis and cirrhosis. To characterise the transcriptomic atlas of liver immune cells involved in schistosoma-associated fibrosis, scRNA-seq analysis was performed on liver samples from fibrotic patients coinfected with *S. japonicum* (SJ) schistosoma and HBV, patients with HBV cirrhosis, and healthy controls [153]. Despite the small sample size, this study revealed that T cells, particularly granzyme B (GZMB)+ T cells, were a major subgroup of liver-resident immune cells and increased in the SJ group. A drastic upregulation was also observed in caveolae-associated protein 2 (CAVIN2)+ macrophages, characterised by the high expression of transforming growth factor B1 (TGFB1) (a master profibrotic gene) in the SJ group [134]. Additionally, in the SJ group, DCs using ring1 and YY1 binding protein (RYBP) as marker genes were the dominant cluster (Fig. 6C). Further analysis suggested that distinct signalling pathways, mainly associated with NK cell-mediated cytotoxicity and antigen processing and presentation, were activated in the SJ group. Although preliminary, this study revealed that liver-resident immune cells with unique cell-marker gene expression and specific signalling pathway activation might deepen our understanding of the mechanism of schistosoma-associated liver fibrosis (Fig. 6C).

### Endotoxemia

In addition to its role in local infections, such as those by hepadna microorganisms, the liver also plays a crucial role in modulating immune defence during acute systemic infections [9, 154, 155]. The liver shifts from an immune tolerant to an immune active state, creating a formidable defence against invading microorganisms as systemic infection takes hold [9, 156]. This defence system relies on a complex network of immune and non-immune cells, including KCs, DCs, T and B lymphocytes, neutrophils, NK cells, hepatocytes, cholangiocytes, and LSECs, which recruit proinflammatory immune cells that produce acute-phase proteins, complement factors, inflammatory cytokines, and cell adhesion molecules to exert immune functions [10–13]. High-throughput single-cell transcriptomics enables us to decipher cell heterogeneity, differentiation, and cell–cell communication during the pathological process occurring in the liver during systemic inflammation. In a study conducted by Cai et al., the cellular landscape of hepatic cells at three different time stages in a “double-hit” endotoxemia mouse model was elucidated. Based on single-cell transcriptomic analyses, the study provided the first evidence that the phenotypic transition occurring in hepatocytes during endotoxemia plays a vital role in the recruitment of circulating monocytes to generate a recruited macrophage population, as well as in the inhibition of T lymphocytes through the CCL2-mediated pathway and upregulation of PD-L1 [157]. This study enhances the comprehension of endotoxemia pathology through a liver-centric perspective and establishes a crucial basis for developing effective therapeutic strategies for acute infections. Human liver DnaJ-like protein (HLJ1), a molecular chaperone belonging to the heat shock protein 40 (HSP40) family, is a potential target for sepsis treatment. ScRNA-seq analysis elucidated the immune profile influenced by HLJ1 in a mouse model challenged with lipopolysaccharides (LPS). HLJ1 plays a critical role in regulating the IL-12/IFN-γ axis-dependent sepsis progression and may serve as a potential molecular target for novel antisepsis or immunomodulatory therapies [158] (Fig. 6D). Additionally, a mouse model of LPS-challenged endotoxemia was used to explore the zonation and spatial heterogeneity of innate immune function in the liver, revealing that NF-KB (p50) activation and c-reactive protein expression in response to endotoxemia are zone-specific [159]. However, this exploration was conducted using traditional RNAscope and immunohistochemistry methods instead of high-throughput spatial transcriptome analysis.

### COVID-19

**Fig. 7:**
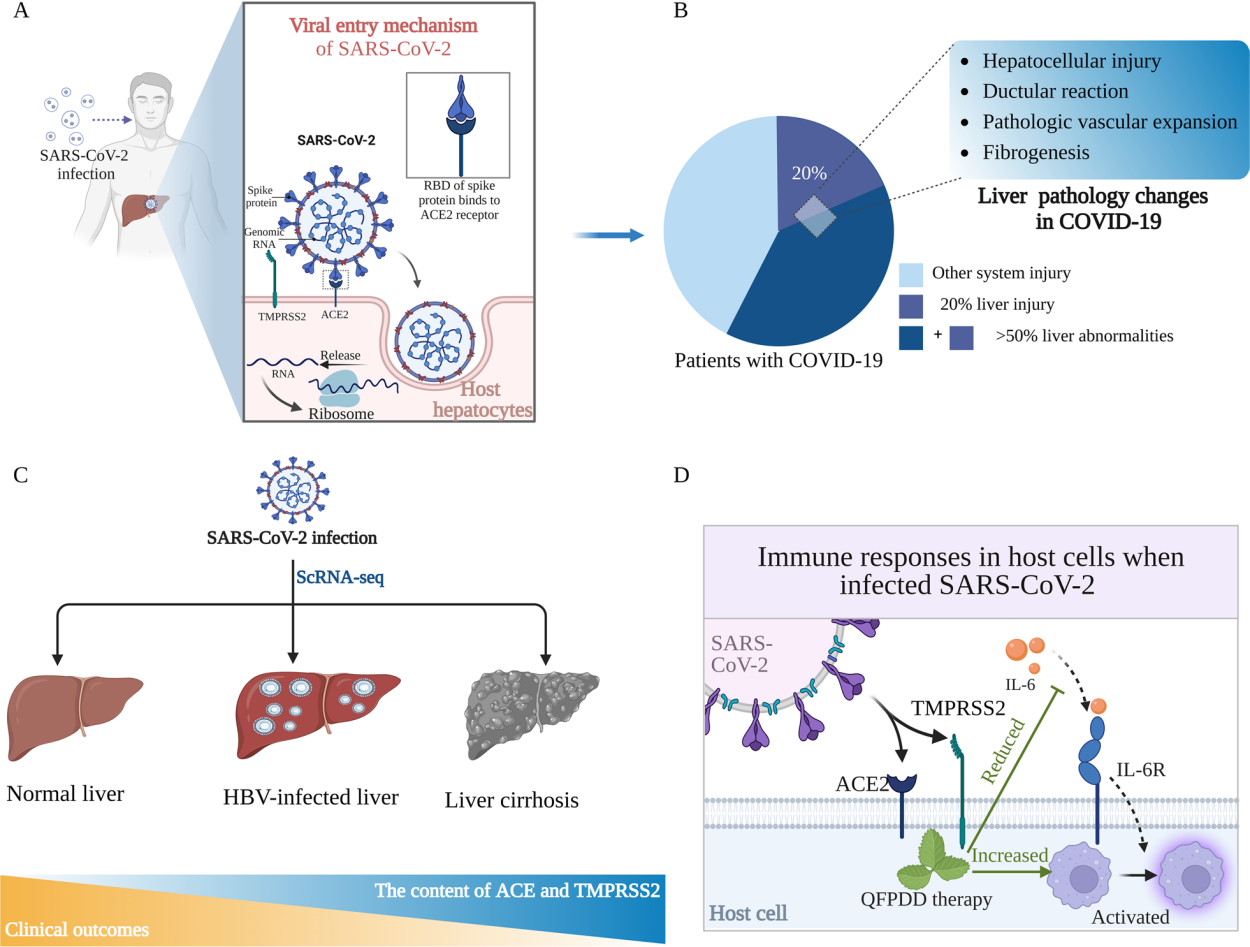
Single-cell perspective of the liver in COVID-19. *ACE2* angiotensin-converting enzyme 2, *COVID-19* Corona Virus Disease 2019, *IL-6* interleukin 6, *IL-6R* interleukin 6 receptor, *QFPDD* Qing-Fei-Pai-Du decoction, *SARS-CoV-2* Severe Acute RespiraTAT3tory Syndrome Coronavirus 2, *ScRNA-seq* single-cell RNA sequencing, *TMPRSS2* transmembrane protease serine 2

Since December 2019, the corona virus disease 2019 (COVID-19) and its pathogen, a novel coronavirus named severe acute respiratory syndrome coronavirus 2 (SARS-CoV-2), have rapidly spread worldwide [160]. The essential factors for SARS-CoV-2 invasion into host cells are angiotensin-converting enzyme 2 (ACE2), a receptor binding to the spike glycoprotein (S) of the virus, and type II transmembrane serine protease (TMPRSS2), which mediates membrane fusion vital to release the viral contents into the infected cell cytosol [161]. Thus, the underlying mechanism of COVID-19-induced organ injury in the human body is related to these two factors (Fig. 7A). Although the lungs are the most frequently affected organ in COVID-19, SARS-CoV-2 can also cause damage to other organs, such as the brain, gut, and liver. Recent studies have shown that liver abnormalities have been observed in more than 50% of patients with COVID-19; in 20% of the patients, the abnormalities subsequently progressed to liver injury [162–166] (Fig. 7B). Comprehensive single-cell/spatial organ atlases of COVID-19 have been generated from various types of patient samples, including livers, using available scRNA-seq and spatial data [166–169]. By performing scRNA-seq and spatial transcriptomic profiling of livers from 17 COVID-19 patients, hepatocytes were identified as positive for SARS-CoV-2 RNA [169]. Further integrated analysis and comparisons with healthy controls revealed massive changes in cellular composition and gene expression in livers affected by COVID-19, mainly in those exhibiting hepatocellular injury, ductular reactions, pathologic vascular expansion, and fibrogenesis (Fig. 7B). This human atlas is fundamental to the investigation and understanding of the liver physiology and pathology in COVID-19 [169]. ACE2 has also been reported to be significantly expressed in liver cholangiocytes, indicating that the liver might be a target organ for SARS-CoV-2 [170]. However, the limitation of the study was its small size (only one liver dataset was analysed), and it did not focus on liver tissue specifically or reported only on ACE2 expression without considering TMPRSS2 expression [171]. Notably, although the percentage of ACE-TMPRSS2 co-expressing hepatocytes was extremely low in the two datasets containing representative hepatocytes [72, 73], it was not entirely absent. Given that the human liver is believed to comprise tens of billions of hepatocytes [172], this extremely low percentage could still endanger millions of hepatocytes.

To further elucidate the specific hepatic cell type implicated in COVID-19 pathophysiology, scRNA-seq analysis was conducted on five representative liver tissue types, including healthy, human foetal, cirrhotic, tumour, and precancerous, comprising over 300,000 single cells. In liver cirrhosis, a population of trophoblast cell surface antigen 2 (TROP2)^+^ liver progenitors was identified as a potential target for viral ingress, suggesting that viral infection of TROP2+ progenitors may significantly impair liver regeneration in cirrhotic patients [173]. Moreover, a study revealed that cirrhotic livers exhibited a higher number of ACE2+ and TMPRSS2+ cells than healthy livers, while HBV-infected livers had the lowest number of ACE2+ and TMPRSS2+ cells [160] (Fig. 7C). This finding may account for the worse clinical outcomes observed in COVID-19 patients with cirrhosis compared to those with viral hepatitis [160]. Using liver organoids derived from human-induced pluripotent stem cells to recapitulate liver pathology following SARS-CoV-2 exposure, robust transcriptomic changes were characterised, and IL-6 signalling was identified as a potential mechanism for liver-mediated activation of circulating macrophages [174] (Fig. 7D).

Clinical studies on potential therapies for COVID-19 are currently in full swing [175, 176]. In China, traditional Chinese medicine (TCM) has played a preventive and therapeutic role in COVID-19 and has been recommended by the latest edition of the COVID-19 guidelines in lightly, moderately, and severely infected patients [177]. The combination of TCM and Western medicine has been shown to reduce inflammation and mitigate multi-organ damage [178]. In a murine model of pneumonia, the therapeutic mechanism of a typical TCM, Qing-Fei-Pai-Du decoction (QFPDD), was systematically investigated using scRNA-seq. The results demonstrated that QFPDD therapy increased peripheral blood lymphocytes (CD4+ and CD8+ T cells, B cells) in mice, reduced the levels of inflammatory cytokines such as IL-6, TNF-α, and IFN-γ, and regulated dysfunctional liver metabolism [179] (Fig. 7D).

The limitation of existing studies is that they cannot determine the impact of vaccination and only reflect on the very early lineages of the virus, as all samples were analysed during the pandemic. To gain more insight into the identity of cell types at risk of SARS-CoV-2 infection, and to uncover potential mechanisms for prospective target identification, CITE-seq that uses antibodies against ACE2 and TMPRSS2 could prove to be a valuable tool.

## Clinical implications

In addition to mapping the healthy and diseased liver to elucidate cellular drivers of liver pathogenesis in infection, single-cell transcriptomics provide an opportunity to enhance the effectiveness of treatment modalities that target the liver or provide additional value for diagnosis and prognosis (Additional file 1: Fig. S2). Prognostic modelling of tumour cell stemness and intratumoural heterogeneity signatures will be relevant in the future for patient stratification of treatments, particularly in liver cancer [180, 181]. Moreover, these unbiased data allow the characterisation of immune features for the molecular classification of patients with liver infection through immunophenotyping, adopting the strategy employed in HCC [182, 183] (Additional file 1: Fig. S2). Deeper comprehension of diseased liver tissue using single-cell multiomics has highlighted diagnostic biomarkers and gene signatures, pathogenic cell subgroups, and potential targetable signalling pathways [184, 185]. To realise this potential, further investigation into preclinical lineage-tracing and gene manipulation research with clinical trials is required to identify druggable targets for future therapeutic applications.

High-throughput single-cell technology has been used in various clinical settings. Single-cell genomics has already been applied to track disease trajectories before and after HCV therapy and tumour immunotherapy, highlighting future clinical applications as a precision medicine approach [130, 135, 186] (Additional file 1: Fig. S2). Moreover, these studies were aimed at investigating whether antiviral therapy facilitates the restoration of T cell responsiveness. Single-cell genomics can also be applied to evaluate the therapeutic efficacy and disease progression in clinical trials of a variety of liver diseases to generate frameworks for genetic, epigenetic, transcriptional, protein, and cellular landscape-based therapy strategies [187] (Additional file 1: Fig. S2). For instance, novel mechanisms and biomarkers of drug resistance have been identified to stratify patients and guide personalised therapeutic decisions via the application of scRNA-seq in clinical trials of multiple myeloma patient cohorts [188, 189]. In the future, patient-specific single-cell analysis of liver biopsies can reveal opportunities for personalised immunology by aiding the evaluation of patient outcomes and treatment effects. The extensive clinical application of single-cell technologies will revolutionise our capacity to track efficacy and tailor treatment strategies for understudied liver pathologies.

### Future directions

In the era of precision medicine, higher-resolution data are needed to identify heterogeneous tissues and complicated diseases, such as microorganism infections. Currently, technologies that aid in the advancement of multiomics single-cell techniques that will allow the characterisation of genetic, epigenetic, transcriptional, proteomic, and metabolic changes in the same cell are being developed. Single-cell multiomics will allow an even more comprehensive understanding of liver biology and disease at a single-cell resolution. Future research should focus on reducing the expense of high-throughput single-cell technologies and identifying molecular, cellular, histological, or radiological deputy biomarkers that facilitate the characterisation and stratification of liver diseases, which will help predict drug efficacy or patient prognosis instead of conducting full single-cell analysis of samples per patient. As high-throughput single-cell techniques are further developed, they will result in a more comprehensive analysis of single cells in healthy and diseased livers, potentially producing novel therapeutic targets. These approaches, which generate more high-dimensional, complicated, and informative resources, necessitate the advancement of cutting-edge bioinformatic algorithms to extract the optimal amount of related information from these integrated datasets. We need to continuously evolve computational analysis pipelines synchronously with methodological advancement to offer new strategies for visualising, analysing, and interpreting single-cell omics datasets (Additional file 1: Fig. S2).

## Conclusion

In summary, high-throughput single-cell and spatial omics are revolutionising our understanding of liver physiology and pathology in healthy and diseased states. These ever-advancing techniques have rapidly and profoundly accelerated the identification of rare- and disease-driving cellular subpopulations within the liver microenvironment. As we continue to become proficient in this technology, expand applications to map initial stages of infection in human tissue, and sample liver tissue longitudinally alongside disease progression, we will unveil novel and effective therapeutic targets and treatment options for patients suffering from various infections.

## Supplementary Information

**Additional file 1: Figure S1.** Single cell perspective of liver in HBV infection. **A** HBV maintenance in hepatocytes. The structure diagram of NTCP is quoted from REF [160]. **B** An unbiased and comprehensive landscape of the intrahepatic immunological characteristic in HBV-infected patients. **C** The roles of Kuffer cellsin HBV-infection. **D** A systematic and distinguishing gene regulatory network of CHB-related PBMCs. **E** The immune microenvironment of infected liver in FVH. **Figure S2.** Clinical implication of high throughout single cell technology.

## Data Availability

Not applicable.

## Abbreviations

ACE2: Angiotensin-converting enzyme 2
COVID-19: Corona virus disease 2019
CdnA: Complementary deoxyribonucleic acid
cccDNA: Covalently closed circular DNA
CRISPR: Clustered regularly interspaced short palindromic repeats
CRISPR-Cas9: CRISPR-CRISPR-associated nuclease 9
CHB: Chronic hepatitis B
CAVIN2: Caveolae-associated protein 2
CITE-seq: Cellular indexing of transcriptomes and epitopes by sequencing
DCs: Dendritic cells
DLL4: Delta-like ligand 4
DNAse: Deoxyribonuclease
DAA: Direct-acting antiviral
EGTA: Ethylene glycol tetraacetic acid
FACS: Fluorescence-activated cell sorting
FVH: Fulminant viral hepatitis
GO: Gene ontology
GSVA: Gene set variation analysis
GZMB: Granzyme B
HBV: Hepatitis B virus
HCV: Hepatitis C virus
HSCs: Hepatic stellate cells
HVGs: Highly variable genes
HCC: Hepatocellular carcinoma
HLJ1: Human liver DnaJ-like protein
HSP40: Heat shock protein 40
IFN: Interferon
ICIs: Inhibitor
JAG1: Jagged 1
JAG2: Jagged 2
KCs: Kupffer cells
KEGG: Kyoto Encyclopedia of Genes and Genomes
LSECs: Liver sinusoidal endothelial cells
LPS: Lipopolysaccharides
MrnA: Messenger ribonucleic acid
miRNAs: MicroRNAs
MHV-3: Hepatitis virus strain-3
NETs: Neutrophil extracellular traps
NK: Natural killer cells
NAFLD: Non-alcoholic fatty liver disease
NC: Normal control
PCR: Polymerase chain reaction
PBMCs: Peripheral blood mononuclear cells
PD-L1: Programmed cell death-ligand 1
*P. vivax*: *Plasmodium vivax*
QFPDD: Qing-Fei-Pai-Du decoction
RAS: Rat sarcoma protein
RCA: Rolling circle amplification
RYBP: Ring1 and YY1 binding protein
ScATAC-seq: Single-cell assay for transposase-accessible chromatin using sequencing
scRNA-seq: Single-cell RNA sequencing
scATAC: Single-cell assay for transposase-accessible chromatin using sequencing
scMT: Single-cell methylome and transcriptome sequencing
snRNA-seq: Single nuclear sequencing
ST: Spatial transcriptomics
STING: Stimulator of interferon genes
SJ: *S. japonicum*
SARS-CoV-2: Severe acute respiratory syndrome coronavirus 2
Tex cells: Exhausted CD8+ T cells
TRM: Tissue-resident memory
TGFB1: Transforming growth factor B1
TMPRSS2: Type II transmembrane serine protease
TROP2: Trophoblast cell surface antigen 2
TCM: Traditional Chinese medicine
UMI: Unique molecular identifier

## References

[1] Paulos CM, Wrzesinski C, Kaiser A, Hinrichs CS, Chieppa M, Cassard L, Palmer DC, Boni A, Muranski P, Yu Z (2007). Microbial translocation augments the function of adoptively transferred self/tumor-specific CD8+ T cells via TLR4 signaling. J Clin Invest.

[2] Wang JY, Ma JP, Nie HY, Zhang XJ, Zhang P, She ZG, Li HL, Ji YX, Cai JJ (2021). Hepatic regulator of G protein signaling 5 ameliorates nonalcoholic fatty liver disease by suppressing transforming growth factor beta-activated kinase 1-c-Jun-N-terminal kinase/p38 signaling. Hepatology.

[3] Thomson AW, Knolle PA (2010). Antigen-presenting cell function in the tolerogenic liver environment. Nat Rev Immunol.

[4] Heymann F, Tacke F (2016). Immunology in the liver—from homeostasis to disease. Nat Rev Gastroenterol Hepatol.

[5] Knolle PA, Thimme R (2014). Hepatic immune regulation and its involvement in viral hepatitis infection. Gastroenterology.

[6] Kubes P, Jenne C (2018). Immune responses in the liver. Annu Rev Immunol.

[7] Guo JL, Li Y, Shan YH, Shu C, Wang F, Wang X, Zheng G, He J, Hu Z, Yang YG (2018). Humanized mice reveal an essential role for human hepatocytes in the development of the liver immune system. Cell Death Dis.

[8] Chung KW, Kim KM, Choi YJ, An HJ, Lee B, Kim DH, Lee EK, Im E, Lee J, Im DS (2017). The critical role played by endotoxin-induced liver autophagy in the maintenance of lipid metabolism during sepsis. Autophagy.

[9] Yan J, Li S, Li SL (2014). The role of the liver in sepsis. Int Rev Immunol.

[10] Mao KR, Baptista AP, Tamoutounour S, Zhuang LN, Bouladoux N, Martins AJ, Huang YF, Gerner MY, Belkaid Y, Germain RN (2018). Innate and adaptive lymphocytes sequentially shape the gut microbiota and lipid metabolism. Nature.

[11] Megahed FAK, Zhou XL, Sun PN (2020). The interactions between HBV and the innate immunity of hepatocytes. Viruses.

[12] Davies SP, Terry LV, Wilkinson AL, Stamataki Z (2020). Cell-in-cell structures in the liver: a tale of four E's. Front Immunol.

[13] Halpern KB, Shenhav R, Massalha H, Toth B, Egozi A, Massasa EE, Medgalia C, David E, Giladi A, Moor AE (2018). Paired-cell sequencing enables spatial gene expression mapping of liver endothelial cells. Nat Biotechnol.

[14] Shapiro E, Biezuner T, Linnarsson S (2013). Single-cell sequencing-based technologies will revolutionize whole-organism science. Nat Rev Genet.

[15] Morrison JK, DeRossi C, Alter IL, Nayar S, Giri M, Zhang C, Cho JH, Chu J (2022). Single-cell transcriptomics reveals conserved cell identities and fibrogenic phenotypes in zebrafish and human liver. Hepatol Commun.

[16] Jovic D, Liang X, Zeng H, Lin L, Xu F, Luo Y (2022). Single-cell RNA sequencing technologies and applications: a brief overview. Clin Transl Med.

[17] Atif J, Thoeni C, Bader GD, McGilvray ID, MacParland SA (2022). Unraveling the complexity of liver disease one cell at a time. Semin Liver Dis.

[18] Andrews TS, Atif J, Liu JC, Perciani CT, Ma XZ, Thoeni C, Slyper M, Eraslan G, Segerstolpe A, Manuel J (2022). Single-cell, single-nucleus, and spatial RNA sequencing of the human liver identifies cholangiocyte and mesenchymal heterogeneity. Hepatol Commun.

[19] Nault R, Fader KA, Bhattacharya S, Zacharewski TR (2021). Single-nuclei RNA sequencing assessment of the hepatic effects of 2,3,7,8-tetrachlorodibenzo-p-dioxin. Cell Mol Gastroenterol Hepatol.

[20] Hildebrandt F, Andersson A, Saarenpaa S, Larsson L, Van Hul N, Kanatani S, Masek J, Ellis E, Barragan A, Mollbrink A (2021). Spatial transcriptomics to define transcriptional patterns of zonation and structural components in the mouse liver. Nat Commun.

[21] Hashimoto S, Shirasaki T, Yamashita T, Iwabuchi S, Suzuki Y, Takamura Y, Ukita Y, Deshimaru S, Okayama T, Ikeo K (2021). DOCK11 and DENND2A play pivotal roles in the maintenance of hepatitis B virus in host cells. PLoS ONE.

[22] Droin C, El Kholtei J, Halpern KB, Hurni C, Rozenberg M, Muvkadi S, Itzkovitz S, Naef F (2021). Space-time logic of liver gene expression at sub-lobular scale. Nat Metab.

[23] De Simone G, Andreata F, Bleriot C, Fumagalli V, Laura C, Garcia-Manteiga JM, Di Lucia P, Gilotto S, Ficht X, De Ponti FF (2021). Identification of a Kupffer cell subset capable of reverting the T cell dysfunction induced by hepatocellular priming. Immunity.

[24] Sun Y, Wu L, Zhong Y, Zhou K, Hou Y, Wang Z, Zhang Z, Xie J, Wang C, Chen D (2021). Single-cell landscape of the ecosystem in early-relapse hepatocellular carcinoma. Cell.

[25] Govaere O, Cockell S, Tiniakos D, Queen R, Younes R, Vacca M, Alexander L, Ravaioli F, Palmer J, Petta S (2020). Transcriptomic profiling across the nonalcoholic fatty liver disease spectrum reveals gene signatures for steatohepatitis and fibrosis. Sci Transl Med.

[26] Hoshida Y, Nijman SMB, Kobayashi M, Chan JA, Brunet JP, Chiang DY, Villanueva A, Newell P, Ikeda K, Hashimoto M (2009). Integrative transcriptome analysis reveals common molecular subclasses of human hepatocellular carcinoma. Can Res.

[27] Boyault S, Rickman DS, de Reynies A, Balabaud C, Rebouissou S, Jeannot E, Herault A, Saric J, Belghiti J, Franco D (2007). Transcriptome classification of HCC is related to gene alterations and to new therapeutic targets. Hepatology.

[28] Yamada S, Nomura S (2020). Review of single-cell RNA sequencing in the heart. Int J Mol Sci.

[29] Gao S (2018). Data analysis in single-cell transcriptome sequencing. Methods Mol Biol.

[30] Lee J, Hyeon DY, Hwang D (2020). Single-cell multiomics: technologies and data analysis methods. Exp Mol Med.

[31] Sklavenitis-Pistofidis R, Getz G, Ghobrial I (2021). Single-cell RNA sequencing: one step closer to the clinic. Nat Med.

[32] van den Brink SC, Sage F, Vertesy A, Spanjaard B, Peterson-Maduro J, Baron CS, Robin C, van Oudenaarden A (2017). Single-cell sequencing reveals dissociation-induced gene expression in tissue subpopulations. Nat Methods.

[33] Adam M, Potter AS, Potter SS (2017). Psychrophilic proteases dramatically reduce single-cell RNA-seq artifacts: a molecular atlas of kidney development. Development.

[34] Dar RD, Razooky BS, Singh A, Trimeloni TV, McCollum JM, Cox CD, Simpson ML, Weinberger LS (2012). Transcriptional burst frequency and burst size are equally modulated across the human genome. Proc Natl Acad Sci USA.

[35] Sarkar A, Stephens M (2021). Separating measurement and expression models clarifies confusion in single-cell RNA sequencing analysis. Nat Genet.

[36] Hsin F, Hsu YC, Tsai YF, Lin SW, Liu HM (2021). The transmembrane serine protease hepsin suppresses type I interferon induction by cleaving STING. Sci Signal.

[37] Thomsen MK, Nandakumar R, Stadler D, Malo A, Valls RM, Wang F, Reinert LS, Dagnaes-Hansen F, Hollensen AK, Mikkelsen JG (2016). Lack of immunological DNA sensing in hepatocytes facilitates hepatitis B virus infection. Hepatology.

[38] Horst AK, Kumashie KG, Neumann K, Diehl L, Tiegs G (2021). Antigen presentation, autoantibody production, and therapeutic targets in autoimmune liver disease. Cell Mol Immunol.

[39] Khnouf R, Shore S, Han CM, Henderson JM, Munro SA, McCaffrey AP, Shintaku H, Santiago JG (2018). Efficient production of on-target reads for small RNA sequencing of single cells using modified adapters. Anal Chem.

[40] Holohan C, Feely N, Li P, Curran G, Lee GU (2022). Role of detergents and nuclease inhibitors in the extraction of RNA from eukaryotic cells in complex matrices. Nanoscale.

[41] Guilliams M, Bonnardel J, Haest B, Vanderborght B, Wagner C, Remmerie A, Bujko A, Martens L, Thoné T, Browaeys R (2022). Spatial proteogenomics reveals distinct and evolutionarily conserved hepatic macrophage niches. Cell.

[42] Xiong X, Kuang H, Ansari S, Liu T, Gong J, Wang S, Zhao XY, Ji Y, Li C, Guo L (2019). Landscape of intercellular crosstalk in healthy and NASH liver revealed by single-cell secretome gene analysis. Mol Cell.

[43] Hines KM, Alvarado G, Chen X, Gatto C, Pokorny A, Alonzo F, Wilkinson BJ, Xu L (2020). Lipidomic and ultrastructural characterization of the cell envelope of staphylococcus aureus grown in the presence of human serum. mSphere.

[44] Stuart T, Butler A, Hoffman P, Hafemeister C, Papalexi E, Mauck WM, Hao Y, Stoeckius M, Smibert P, Satija R (2019). Comprehensive integration of single-cell data. Cell.

[45] Si-Tayeb K, Lemaigre FP, Duncan SA (2010). Organogenesis and development of the liver. Dev Cell.

[46] Banales JM, Huebert RC, Karlsen T, Strazzabosco M, LaRusso NF, Gores GJ (2019). Cholangiocyte pathobiology. Nat Rev Gastroenterol Hepatol.

[47] Sun HJ, Chen J, Ni B, Yang X, Wu YZ (2015). Recent advances and current issues in single-cell sequencing of tumors. Cancer Lett.

[48] Li H (2021). Single-cell RNA sequencing in Drosophila: technologies and applications. Wiley Interdiscip Rev Dev Biol.

[49] Macaulay IC, Voet T (2014). Single cell genomics: advances and future perspectives. PLoS Genet.

[50] Hwang B, Lee JH, Bang D (2018). Single-cell RNA sequencing technologies and bioinformatics pipelines. Exp Mol Med.

[51] Nassar SF, Raddassi K, Wu T (2021). Single-cell multiomics analysis for drug discovery. Metabolites.

[52] Gao C, Zhang M, Chen L (2020). The comparison of two single-cell sequencing platforms: BD rhapsody and 10x Genomics chromium. Curr Genom.

[53] Ziegenhain C, Vieth B, Parekh S, Reinius B, Guillaumet-Adkins A, Smets M, Leonhardt H, Heyn H, Hellmann I, Enard W (2017). Comparative analysis of single-cell RNA sequencing methods. Mol Cell.

[54] Fan HC, Fu GK, Fodor SP (2015). Expression profiling. Combinatorial labeling of single cells for gene expression cytometry. Science.

[55] Hughes TK, Wadsworth MH, Gierahn TM, Do T, Weiss D, Andrade PR, Ma F, de Andrade Silva BJ, Shao S, Tsoi LC (2020). Second-strand synthesis-based massively parallel scRNA-seq reveals cellular states and molecular features of human inflammatory skin pathologies. Immunity.

[56] Valihrach L, Androvic P, Kubista M (2018). Platforms for single-cell collection and analysis. Int J Mol Sci.

[57] Hwang B, Lee JH, Bang D (2021). Single-cell RNA sequencing technologies and bioinformatics pipelines (vol 50, pg 1, 2018). Exp Mol Med.

[58] Chen G, Ning BT, Shi TL (2019). Single-cell RNA-seq technologies and related computational data analysis. Front Genet.

[59] Poch T, Krause J, Casar C, Liwinski T, Glau L, Kaufmann M, Ahrenstorf AE, Hess LU, Ziegler AE, Martrus G (2021). Single-cell atlas of hepatic T cells reveals expansion of liver-resident naive-like CD4(+) T cells in primary sclerosing cholangitis. J Hepatol.

[60] Datlinger P, Rendeiro AF, Schmidl C, Krausgruber T, Traxler P, Klughammer J, Schuster LC, Kuchler A, Alpar D, Bock C (2017). Pooled CRISPR screening with single-cell transcriptome readout. Nat Methods.

[61] Gawad C, Koh W, Quake SR (2016). Single-cell genome sequencing: current state of the science. Nat Rev Genet.

[62] Ranzoni AM, Tangherloni A, Berest I, Riva SG, Myers B, Strzelecka PM, Xu J, Panada E, Mohorianu I, Zaugg JB (2021). Integrative single-cell RNA-seq and ATAC-seq analysis of human developmental hematopoiesis. Cell Stem Cell.

[63] Hu Y, Huang K, An Q, Du G, Hu G, Xue J, Zhu X, Wang CY, Xue Z, Fan G (2016). Simultaneous profiling of transcriptome and DNA methylome from a single cell. Genome Biol.

[64] Hu Y, An Q, Guo Y, Zhong J, Fan S, Rao P, Liu X, Liu Y, Fan G (2019). Simultaneous profiling of mRNA transcriptome and DNA methylome from a single cell. Methods Mol Biol.

[65] Golomb SM, Guldner IH, Zhao A, Wang Q, Palakurthi B, Aleksandrovic EA, Lopez JA, Lee SW, Yang K, Zhang S (2020). Multi-modal single-cell analysis reveals brain immune landscape plasticity during aging and gut microbiota dysbiosis. Cell Rep.

[66] Stoeckius M, Hafemeister C, Stephenson W, Houck-Loomis B, Chattopadhyay PK, Swerdlow H, Satija R, Smibert P (2017). Simultaneous epitope and transcriptome measurement in single cells. Nat Methods.

[67] Zheng W, Zhao S, Yin Y, Zhang H, Needham DM, Evans ED, Dai CL, Lu PJ, Alm EJ, Weitz DA (2022). High-throughput, single-microbe genomics with strain resolution, applied to a human gut microbiome. Science.

[68] Baccin C, Al-Sabah J, Velten L, Helbling PM, Grunschlager F, Hernandez-Malmierca P, Nombela-Arrieta C, Steinmetz LM, Trumpp A, Haas S (2020). Combined single-cell and spatial transcriptomics reveal the molecular, cellular and spatial bone marrow niche organization. Nat Cell Biol.

[69] Wu H, Kirita Y, Donnelly EL, Humphreys BD (2019). Advantages of single-nucleus over single-cell RNA sequencing of adult kidney: rare Cell types and novel cell states revealed in fibrosis. J Am Soc Nephrol.

[70] Slyper M, Porter CBM, Ashenberg O, Waldman J, Drokhlyansky E, Wakiro I, Smillie C, Smith-Rosario G, Wu J, Dionne D (2020). A single-cell and single-nucleus RNA-Seq toolbox for fresh and frozen human tumors. Nat Med.

[71] Rodrigues PM, Banales JM (2021). Characterizing the heterogeneity of liver cell populations under a NASH-related hepatotoxicant using single-nuclei RNA sequencing. Cell Mol Gastroenterol Hepatol.

[72] Aizarani N, Saviano A, Sagar, Mailly L, Durand S, Herman JS, Pessaux P, Baumert TF, Grun D (2019). A human liver cell atlas reveals heterogeneity and epithelial progenitors. Nature.

[73] MacParland SA, Liu JC, Ma XZ, Innes BT, Bartczak AM, Gage BK, Manuel J, Khuu N, Echeverri J, Linares I (2018). Single cell RNA sequencing of human liver reveals distinct intrahepatic macrophage populations. Nat Commun.

[74] Ben-Moshe S, Itzkovitz S (2019). Spatial heterogeneity in the mammalian liver. Nat Rev Gastroenterol Hepatol.

[75] Halpern KB, Shenhav R, Matcovitch-Natan O, Toth B, Lemze D, Golan M, Massasa EE, Baydatch S, Landen S, Moor AE (2017). Single-cell spatial reconstruction reveals global division of labour in the mammalian liver. Nature.

[76] Chu AL, Schilling JD, King KR, Feldstein AE (2021). The power of single-cell analysis for the study of liver pathobiology. Hepatology.

[77] Saito K, Negishi M, James SE (2013). Sexual dimorphisms in zonal gene expression in mouse liver. Biochem Biophys Res Commun.

[78] Braeuning A, Ittrich C, Kohle C, Hailfinger S, Bonin M, Buchmann A, Schwarz M (2006). Differential gene expression in periportal and perivenous mouse hepatocytes. FEBS J.

[79] Qian X, Harris KD, Hauling T, Nicoloutsopoulos D, Munoz-Manchado AB, Skene N, Hjerling-Leffler J, Nilsson M (2020). Probabilistic cell typing enables fine mapping of closely related cell types in situ. Nat Methods.

[80] Tran HTN, Ang KS, Chevrier M, Zhang X, Lee NYS, Goh M, Chen J (2020). A benchmark of batch-effect correction methods for single-cell RNA sequencing data. Genome Biol.

[81] Karaiskos N, Wahle P, Alles J, Boltengagen A, Ayoub S, Kipar C, Kocks C, Rajewsky N, Zinzen RP (2017). The Drosophila embryo at single-cell transcriptome resolution. Science.

[82] Armingol E, Officer A, Harismendy O, Lewis NE (2021). Deciphering cell–cell interactions and communication from gene expression. Nat Rev Genet.

[83] Longo SK, Guo MG, Ji AL, Khavari PA (2021). Integrating single-cell and spatial transcriptomics to elucidate intercellular tissue dynamics. Nat Rev Genet.

[84] Godoy P, Hewitt NJ, Albrecht U, Andersen ME, Ansari N, Bhattacharya S, Bode JG, Bolleyn J, Borner C, Bottger J (2013). Recent advances in 2D and 3D in vitro systems using primary hepatocytes, alternative hepatocyte sources and non-parenchymal liver cells and their use in investigating mechanisms of hepatotoxicity, cell signaling and ADME. Arch Toxicol.

[85] Cheng ML, Nakib D, Perciani CT, MacParland SA (2021). The immune niche of the liver. Clin Sci (Lond).

[86] Hilscher MB, Shah VH (2020). Small but mighty: platelets in NASH and other chronic liver diseases. Hepatology.

[87] Zhao JJ, Zhang SY, Liu Y, He XM, Qu MM, Xu G, Wang HB, Huang M, Pan J, Liu ZW (2020). Single-cell RNA sequencing reveals the heterogeneity of liver-resident immune cells in human. Cell Discov.

[88] Tamburini BAJ, Finlon JM, Gillen AE, Kriss MS, Riemondy KA, Fu R, Schuyler RP, Hesselberth JR, Rosen HR, Burchill MA (2019). Chronic liver disease in humans causes expansion and differentiation of liver lymphatic endothelial cells. Front Immunol.

[89] Segal JM, Kent D, Wesche DJ, Ng SS, Serra M, Oules B, Kar G, Emerton G, Blackford SJI, Darmanis S (2019). Single cell analysis of human foetal liver captures the transcriptional profile of hepatobiliary hybrid progenitors. Nat Commun.

[90] Ramachandran P, Dobie R, Wilson-Kanamori JR, Dora EF, Henderson BEP, Luu NT, Portman JR, Matchett KP, Brice M, Marwick JA (2019). Resolving the fibrotic niche of human liver cirrhosis at single-cell level. Nature.

[91] Brancale J, Vilarinho S (2021). A single cell gene expression atlas of 28 human livers. J Hepatol.

[92] Browaeys R, Saelens W, Saeys Y (2020). NicheNet: modeling intercellular communication by linking ligands to target genes. Nat Methods.

[93] Vento-Tormo R, Efremova M, Botting RA, Turco MY, Vento-Termo M, Meyer KB, Park JE, Stephenson E, Polanski K, Goncalves A (2018). Single-cell reconstruction of the early maternal-fetal interface in humans. Nature.

[94] Cohen M, Giladi A, Gorki AD, Solodkin DG, Zada M, Hladik A, Miklosi A, Salame TM, Halpern KB, David E (2018). Lung single-cell signaling interaction map reveals basophil role in macrophage imprinting. Cell.

[95] Llewellyn HP, Arat S, Gao J, Wen J, Xia S, Kalabat D, Oziolor E, Virgen-Slane R, Affolter T, Ji C (2021). T cells and monocyte-derived myeloid cells mediate immunotherapy-related hepatitis in a mouse model. J Hepatol.

[96] Ramilowski JA, Goldberg T, Harshbarger J, Kloppmann E, Lizio M, Satagopam VP, Itoh M, Kawaji H, Carninci P, Rost B (2015). A draft network of ligand-receptor-mediated multicellular signalling in human. Nat Commun.

[97] Hoehme S, Brulport M, Bauer A, Bedawy E, Schormann W, Hermes M, Puppe V, Gebhardt R, Zellmer S, Schwarz M (2010). Prediction and validation of cell alignment along microvessels as order principle to restore tissue architecture in liver regeneration. Proc Natl Acad Sci USA.

[98] Teutsch HF (2005). The modular microarchitecture of human liver. Hepatology.

[99] Gebhardt R (1992). Metabolic zonation of the liver: regulation and implications for liver function. Pharmacol Ther.

[100] Halpern KB, Shenhav R, Matcovitch-Natan O, Toth B, Lemze D, Golan M, Massasa EE, Baydatch S, Landen S, Moor AE et al. Single-cell spatial reconstruction reveals global division of labour in the mammalian liver (vol 542, pg 352, 2017)*.* Nature. 2017;543(7647). 10.1038/nature21065 PMC5321580 28166538

[101] Planas-Paz L, Orsini V, Boulter L, Calabrese D, Pikiolek M, Nigsch F, Xie Y, Roma G, Donovan A, Marti P (2016). The RSPO-LGR4/5-ZNRF3/RNF43 module controls liver zonation and size (vol 18, pg 467, 2016). Nat Cell Biol.

[102] Vickovic S, Eraslan G, Salmen F, Klughammer J, Stenbeck L, Schapiro D, Aijo T, Bonneau R, Bergenstrahle L, Navarro JF (2019). High-definition spatial transcriptomics for in situ tissue profiling. Nat Methods.

[103] Wang X, Allen WE, Wright MA, Sylwestrak EL, Samusik N, Vesuna S, Evans K, Liu C, Ramakrishnan C, Liu J (2018). Three-dimensional intact-tissue sequencing of single-cell transcriptional states. Science.

[104] Ben-Moshe S, Shapira Y, Moor AE, Manco R, Veg T, Halpern KB, Itzkovitz S (2019). Spatial sorting enables comprehensive characterization of liver zonation. Nat Metab.

[105] Dobie R, Wilson-Kanamori JR, Henderson BEP, Smith JR, Matchett KP, Portman JR, Wallenborg K, Picelli S, Zagorska A, Pendem SV (2019). Single-cell transcriptomics uncovers zonation of function in the mesenchyme during liver fibrosis. Cell Rep.

[106] Ivanovska I, Ball AS, Diaz RL, Magnus JF, Kibukawa M, Schelter JM, Kobayashi SV, Lim L, Burchard J, Jackson AL (2008). MicroRNAs in the miR-106b family regulate p21/CDKN1A and promote cell cycle progression. Mol Cell Biol.

[107] Kota J, Chivukula RR, O'Donnell KA, Wentzel EA, Montgomery CL, Hwang HW, Chang TC, Vivekanandan P, Torbenson M, Clark KR (2009). Therapeutic microRNA delivery suppresses tumorigenesis in a murine liver cancer model. Cell.

[108] Jopling CL, Yi M, Lancaster AM, Lemon SM, Sarnow P (2005). Modulation of hepatitis C virus RNA abundance by a liver-specific microRNA. Science.

[109] Roderburg C, Urban GW, Bettermann K, Vucur M, Zimmermann H, Schmidt S, Janssen J, Koppe C, Knolle P, Castoldi M (2011). Micro-RNA profiling reveals a role for miR-29 in human and murine liver fibrosis. Hepatology.

[110] Holzhutter HG, Drasdo D, Preusser T, Lippert J, Henney AM (2012). The virtual liver: a multidisciplinary, multilevel challenge for systems biology. Wiley Interdiscip Rev Syst Biol Med.

[111] Roehlen N, Crouchet E, Baumert TF (2020). Liver fibrosis: mechanistic concepts and therapeutic perspectives. Cells.

[112] Lozano R, Naghavi M, Foreman K, AlMazroa MA, Memish ZA (2013). Global and regional mortality from 235 causes of death for 20 age groups in 1990 and 2010: a systematic analysis for the Global Burden of Disease Study 2010 (vol 380, pg 2095, 2012). Lancet.

[113] Yuen MF, Chen DS, Dusheiko GM, Janssen HLA, Lau DTY, Locarnini SA, Peters MG, Lai CL (2018). Hepatitis B virus infection. Nat Rev Dis Prim.

[114] Zhang C, Li J, Cheng Y, Meng F, Song JW, Fan X, Fan H, Li J, Fu YL, Zhou MJ (2022). Single-cell RNA sequencing reveals intrahepatic and peripheral immune characteristics related to disease phases in HBV-infected patients. Gut.

[115] Sacks D, Baxter B, Campbell BCV, Carpenter JS, Cognard C, Dippel D, From the American Association of Neurological Surgeons (AANS), American Society of Neuroradiology (ASNR), Cardiovascular and Interventional Radiology Society of Europe (CIRSE), Canadian Interventional Radiology Association (CIRA), Congress of Neurological Surgeons (CNS), European Society of Minimally Invasive Neurological Therapy (ESMINT), European Society of Neuroradiology (ESNR), European Stroke Organization (ESO), Society for Cardiovascular Angiography and Interventions (SCAI), Society of Interventional Radiology (SIR), Society of NeuroInterventional Surgery (SNIS), World Stroke Organization (WSO) (2018). Multisociety consensus quality improvement revised consensus statement for endovascular therapy of acute ischemic stroke. Int J Stroke.

[116] Le C, Liu Y, Lopez-Orozco J, Joyce MA, Le XC, Tyrrell DL (2021). CRISPR technique incorporated with single-cell RNA sequencing for studying hepatitis B infection. Anal Chem.

[117] Chen X, Shen Y, Draper W, Buenrostro JD, Litzenburger U, Cho SW, Satpathy AT, Carter AC, Ghosh RP, East-Seletsky A (2016). ATAC-see reveals the accessible genome by transposase-mediated imaging and sequencing. Nat Methods.

[118] Thurman RE, Rynes E, Humbert R, Vierstra J, Maurano MT, Haugen E, Sheffield NC, Stergachis AB, Wang H, Vernot B (2012). The accessible chromatin landscape of the human genome. Nature.

[119] Xu H, Yu H, Zheng F, Zhang C, Cai W, Zhang X, Tang D, Dai Y (2022). Analyzing the gene regulatory network in hepatitis B patients by single-cell ATAC sequencing. Clin Rheumatol.

[120] Li X, Gao Q, Wu W, Hai S, Hu J, You J, Huang D, Wang H, Wu D, Han M (2022). FGL2-MCOLN3-autophagy axis-triggered neutrophil extracellular traps exacerbate liver injury in fulminant viral hepatitis. Cell Mol Gastroenterol Hepatol.

[121] Wang X, Ning Q (2014). Immune mediated liver failure. EXCLI J.

[122] Neuveut C, Wei Y, Buendia MA (2010). Mechanisms of HBV-related hepatocarcinogenesis. J Hepatol.

[123] Suhail M, Abdel-Hafiz H, Ali A, Fatima K, Damanhouri GA, Azhar E, Chaudhary AG, Qadri I (2014). Potential mechanisms of hepatitis B virus induced liver injury. World J Gastroenterol.

[124] Duan M, Hao J, Cui S, Worthley DL, Zhang S, Wang Z, Shi J, Liu L, Wang X, Ke A (2018). Diverse modes of clonal evolution in HBV-related hepatocellular carcinoma revealed by single-cell genome sequencing. Cell Res.

[125] Shoukry NH, Hepatitis C (2018). Vaccines, antibodies, and T cells. Front Immunol.

[126] Panel A-IHG (2018). Hepatitis C guidance 2018 update: AASLD-IDSA recommendations for testing, managing, and treating hepatitis C virus infection. Clin Infect Dis.

[127] Sandmann L, Schulte B, Manns MP, Maasoumy B (2019). Treatment of chronic hepatitis C: efficacy, side effects and complications. Visc Med.

[128] Martin B, Hennecke N, Lohmann V, Kayser A, Neumann-Haefelin C, Kukolj G, Bocher WO, Thimme R (2014). Restoration of HCV-specific CD8+ T cell function by interferon-free therapy. J Hepatol.

[129] Emmanuel B, El-Kamary SS, Magder LS, Stafford KA, Charurat ME, Poonia B, Chairez C, McLaughlin M, Hadigan C, Masur H (2019). Immunological recovery in T-cell activation after sustained virologic response among HIV positive and HIV negative chronic Hepatitis C patients. Hepatol Int.

[130] Burchill MA, Salomon MP, Golden-Mason L, Wieland A, Maretti-Mira AC, Gale M, Rosen HR (2021). Single-cell transcriptomic analyses of T cells in chronic HCV-infected patients dominated by DAA-induced interferon signaling changes. PLoS Pathog.

[131] Blank CU, Haining WN, Held W, Hogan PG, Kallies A, Lugli E, Lynn RC, Philip M, Rao A, Restifo NP (2019). Defining ‘T cell exhaustion’. Nat Rev Immunol.

[132] Gallimore A, Glithero A, Godkin A, Tissot AC, Pluckthun A, Elliott T, Hengartner H, Zinkernagel R (1998). Induction and exhaustion of lymphocytic choriomeningitis virus-specific cytotoxic T lymphocytes visualized using soluble tetrameric major histocompatibility complex class I-peptide complexes. J Exp Med.

[133] Moskophidis D, Lechner F, Pircher H, Zinkernagel RM (1993). Virus persistence in acutely infected immunocompetent mice by exhaustion of antiviral cytotoxic effector T cells. Nature.

[134] Zajac AJ, Blattman JN, Murali-Krishna K, Sourdive DJ, Suresh M, Altman JD, Ahmed R (1998). Viral immune evasion due to persistence of activated T cells without effector function. J Exp Med.

[135] Hensel N, Gu Z, Sagar, Wieland D, Jechow K, Kemming J, Llewellyn-Lacey S, Gostick E, Sogukpinar O, Emmerich F (2021). Memory-like HCV-specific CD8(+) T cells retain a molecular scar after cure of chronic HCV infection. Nat Immunol.

[136] Cui A, Li B, Wallace MS, Gonye ALK, Oetheimer C, Patel H, Tonnerre P, Holmes JA, Lieb D, Yao BS (2023). Single-cell atlas of the liver myeloid compartment before and after cure of chronic viral hepatitis. J Hepatol.

[137] Organization WH. World malaria report 2021 in World malaria report 2021; 2021.

[138] McDonald J, Merrick CJ (2022). DNA replication dynamics during erythrocytic schizogony in the malaria parasites *Plasmodium falciparum* and *Plasmodium knowlesi*. PLoS Pathog.

[139] Krotoski WA, Collins WE, Bray RS, Garnham PC, Cogswell FB, Gwadz RW, Killick-Kendrick R, Wolf R, Sinden R, Koontz LC (1982). Demonstration of hypnozoites in sporozoite-transmitted *Plasmodium vivax* infection. Am J Trop Med Hyg.

[140] White MT, Walker P, Karl S, Hetzel MW, Freeman T, Waltmann A, Laman M, Robinson LJ, Ghani A, Mueller I (2018). Mathematical modelling of the impact of expanding levels of malaria control interventions on *Plasmodium vivax*. Nat Commun.

[141] Gural N, Mancio-Silva L, Miller AB, Galstian A, Butty VL, Levine SS, Patrapuvich R, Desai SP, Mikolajczak SA, Kappe SHI (2018). In vitro culture, drug sensitivity, and transcriptome of *Plasmodium vivax* hypnozoites. Cell Host Microbe.

[142] Mancio-Silva L, Gural N, Real E, Wadsworth MH, Butty VL, March S, Nerurkar N, Hughes TK, Roobsoong W, Fleming HE (2022). A single-cell liver atlas of *Plasmodium vivax* infection. Cell Host Microbe.

[143] Gierahn TM, Wadsworth MH, Hughes TK, Bryson BD, Butler A, Satija R, Fortune S, Love JC, Shalek AK (2017). Seq-Well: portable, low-cost RNA sequencing of single cells at high throughput. Nat Methods.

[144] Ruberto AA, Maher SP, Vantaux A, Joyner CJ, Bourke C, Balan B, Jex A, Mueller I, Witkowski B, Kyle DE (2022). Single-cell RNA profiling of *Plasmodium vivax*-infected hepatocytes reveals parasite- and host- specific transcriptomic signatures and therapeutic targets. Front Cell Infect Microbiol.

[145] Ng S, March S, Galstian A, Hanson K, Carvalho T, Mota MM, Bhatia SN (2014). Hypoxia promotes liver-stage malaria infection in primary human hepatocytes in vitro. Dis Model Mech.

[146] Yang ASP, van Waardenburg YM, van de Vegte-Bolmer M, van Gemert GA, Graumans W, de Wilt JHW, Sauerwein RW (2021). Zonal human hepatocytes are differentially permissive to *Plasmodium falciparum* malaria parasites. EMBO J.

[147] Afriat A, Zuzarte-Luis V, Bahar Halpern K, Buchauer L, Marques S, Chora AF, Lahree A, Amit I, Mota MM, Itzkovitz S (2022). A spatiotemporally resolved single-cell atlas of the *Plasmodium* liver stage. Nature.

[148] Bahar Halpern K, Tanami S, Landen S, Chapal M, Szlak L, Hutzler A, Nizhberg A, Itzkovitz S (2015). Bursty gene expression in the intact mammalian liver. Mol Cell.

[149] Noe A, Datoo MS, Flaxman A, Husainy MA, Jenkin D, Bellamy D, Makinson RA, Morter R, Ramos Lopez F, Sheridan J (2022). Deep Immune phenotyping and single-cell transcriptomics allow identification of circulating TRM-like cells which correlate with liver-stage immunity and vaccine-induced protection from malaria. Front Immunol.

[150] Arnaud V, Li J, Wang Y, Fu X, Mengzhi S, Luo X, Hou X, Dessein H, Jie Z, Xin-Ling Y (2008). Regulatory role of interleukin-10 and interferon-gamma in severe hepatic central and peripheral fibrosis in humans infected with Schistosoma japonicum. J Infect Dis.

[151] Huang P, Zhou M, Cheng S, Hu Y, Gao M, Ma Y, Limpanont Y, Zhou H, Dekumyoy P, Cheng Y (2020). Myricetin possesses anthelmintic activity and attenuates hepatic fibrosis via modulating TGFbeta1 and Akt signaling and shifting Th1/Th2 balance in *Schistosoma japonicum*-infected mice. Front Immunol.

[152] Warren KS, Mahmoud AA, Cummings P, Murphy DJ, Houser HB (1974). *Schistosomiasis mansoni* in Yemeni in California: duration of infection, presence of disease, herapeutic management. Am J Trop Med Hyg.

[153] Zhang Y, Li J, Li H, Zhou Z, Guo C, Jiang J, Ming Y (2021). A preliminary investigation into the immune cell landscape of schistosome-associated liver fibrosis in humans. Immunol Cell Biol.

[154] Woźnica EA, Inglot M, Woźnica RK, Łysenko L (2018). Liver dysfunction in sepsis. Adv Clin Exp Med.

[155] Gc C (1997). Liver involvement in systemic infection. Eur J Gastroenterol Hepatol.

[156] Strnad P, Tacke F, Koch A, Trautwein C (2017). Liver—guardian, modifier and target of sepsis. Nat Rev Gastroenterol Hepatol.

[157] Sun X, Wu J, Liu L, Chen Y, Tang Y, Liu S, Chen H, Jiang Y, Liu Y, Yuan H (2022). Transcriptional switch of hepatocytes initiates macrophage recruitment and T-cell suppression in endotoxemia. J Hepatol.

[158] Luo WJ, Yu SL, Chang CC, Chien MH, Chang YL, Liao KM, Lin PC, Chung KP, Chuang YH, Chen JJW (2022). HLJ1 amplifies endotoxin-induced sepsis severity by promoting IL-12 heterodimerization in macrophages. Elife.

[159] McCarthy WC, Sherlock LG, Grayck MR, Zheng L, Lacayo OA, Solar M, Orlicky DJ, Dobrinskikh E, Wright CJ (2023). Innate immune zonation in the liver: NF-kappaB (p50) activation and C-reactive protein expression in response to endotoxemia are zone specific. J Immunol.

[160] Shao J, Liang Y, Li Y, Ding R, Zhu M, You W, Wang Z, Huang B, Wu M, Zhang T (2021). Implications of liver injury in risk-stratification and management of patients with COVID-19. Hepatol Int.

[161] Hoffmann M, Kleine-Weber H, Schroeder S, Krüger N, Herrler T, Erichsen S, Schiergens TS, Herrler G, Wu N-H, Nitsche A (2020). SARS-CoV-2 cell entry depends on ACE2 and TMPRSS2 and is blocked by a clinically proven protease inhibitor. Cell.

[162] Huang C, Wang Y, Li X, Ren L, Zhao J, Hu Y, Zhang L, Fan G, Xu J, Gu X (2020). Clinical features of patients infected with 2019 novel coronavirus in Wuhan. China Lancet.

[163] Cai Q, Huang D, Yu H, Zhu Z, Xia Z, Su Y, Li Z, Zhou G, Gou J, Qu J (2020). COVID-19: abnormal liver function tests. J Hepatol.

[164] Zhang C, Shi L, Wang FS (2020). Liver injury in COVID-19: management and challenges. Lancet Gastroenterol Hepatol.

[165] Fu Y, Zhu R, Bai T, Han P, He Q, Jing M, Xiong X, Zhao X, Quan R, Chen C (2020). Clinical features of patients infected with coronavirus disease 2019 with elevated liver biochemistries: a multicenter, retrospective study. Hepatology.

[166] Dey A, Sen S, Maulik U (2021). Unveiling COVID-19-associated organ-specific cell types and cell-specific pathway cascade. Brief Bioinform.

[167] Delorey TM, Ziegler CGK, Heimberg G, Normand R, Yang Y, Segerstolpe A, Abbondanza D, Fleming SJ, Subramanian A, Montoro DT (2021). COVID-19 tissue atlases reveal SARS-CoV-2 pathology and cellular targets. Nature.

[168] Ilieva M, Tschaikowski M, Vandin A, Uchida S (2022). The current status of gene expression profilings in COVID-19 patients. Clin Transl Discov.

[169] Pita-Juarez Y, Karagkouni D, Kalavros N, Melms JC, Niezen S, Delorey TM, Essene AL, Brook OR, Pant D, Skelton-Badlani D (2022). A single-nucleus and spatial transcriptomic atlas of the COVID-19 liver reveals topological, functional, and regenerative organ disruption in patients. bioRxiv.

[170] Qi F, Qian S, Zhang S, Zhang Z (2020). Single cell RNA sequencing of 13 human tissues identify cell types and receptors of human coronaviruses. Biochem Biophys Res Commun.

[171] De Smet V, Verhulst S, van Grunsven LA (2020). Single cell RNA sequencing analysis did not predict hepatocyte infection by SARS-CoV-2. J Hepatol.

[172] Bianconi E, Piovesan A, Facchin F, Beraudi A, Casadei R, Frabetti F, Vitale L, Pelleri MC, Tassani S, Piva F (2013). An estimation of the number of cells in the human body. Ann Hum Biol.

[173] Seow JJW, Pai R, Mishra A, Shepherdson E, Lim TKH, Goh BKP, Chan JKY, Chow PKH, Ginhoux F, DasGupta R (2021). Single-cell RNA-seq reveals angiotensin-converting enzyme 2 and transmembrane serine protease 2 expression in TROP2(+) liver progenitor cells: implications in coronavirus disease 2019-associated liver dysfunction. Front Med (Lausanne).

[174] Richards A, Friesen M, Khalil A, Barrasa MI, Gehrke L, Jaenisch R (2022). SARS-CoV-2 infection of human pluripotent stem cell-derived liver organoids reveals potential mechanisms of liver pathology. iScience.

[175] Shi N, Liu B, Liang N, Ma Y, Ge Y, Yi H, Wo H, Gu H, Kuang Y, Tang S (2020). Association between early treatment with Qingfei Paidu decoction and favorable clinical outcomes in patients with COVID-19: a retrospective multicenter cohort study. Pharmacol Res.

[176] Shin MD, Shukla S, Chung YH, Beiss V, Chan SK, Ortega-Rivera OA, Wirth DM, Chen A, Sack M, Pokorski JK (2020). COVID-19 vaccine development and a potential nanomaterial path forward. Nat Nanotechnol.

[177] National Health Commission of the People’s Republic of China. The new coronary virus pneumonia diagnosis and treatment plan (Trial Ninth Edition)*.* http://www.nhc.gov.cn/yzygj/s7653p/202203/b74ade1ba4494583805a3d2e40093d88/files/ef09aa4070244620b010951b088b8a27.pdf. Accessed 15 Mar 2022.

[178] Xin S, Cheng X, Zhu B, Liao X, Yang F, Song L, Shi Y, Guan X, Su R, Wang J (2020). Clinical retrospective study on the efficacy of Qingfei Paidu decoction combined with Western medicine for COVID-19 treatment. Biomed Pharmacother.

[179] Tian S, Zheng N, Zu X, Wu G, Zhong J, Zhang J, Sheng L, Liu W, Wang C, Ge G (2022). Integrated hepatic single-cell RNA sequencing and untargeted metabolomics reveals the immune and metabolic modulation of Qing-Fei-Pai-Du decoction in mice with coronavirus-induced pneumonia. Phytomedicine.

[180] Su M, Qiao KY, Xie XL, Zhu XY, Gao FL, Li CJ, Zhao DQ (2020). Development of a prognostic signature based on single-cell RNA sequencing data of immune cells in intrahepatic cholangiocarcinoma. Front Genet.

[181] Wang H, Yu S, Cai Q, Ma D, Yang L, Zhao J, Jiang L, Zhang X, Yu Z (2021). The prognostic model based on tumor cell evolution trajectory reveals a different risk group of hepatocellular carcinoma. Front Cell Dev Biol.

[182] Zhang Q, Lou Y, Yang J, Wang J, Feng J, Zhao Y, Wang L, Huang X, Fu Q, Ye M (2019). Integrated multiomic analysis reveals comprehensive tumour heterogeneity and novel immunophenotypic classification in hepatocellular carcinomas. Gut.

[183] Ho DW-H, Tsui Y-M, Chan L-K, Sze KM-F, Zhang X, Cheu JW-S, Chiu Y-T, Lee JM-F, Chan AC-Y, Cheung ET-Y (2021). Single-cell RNA sequencing shows the immunosuppressive landscape and tumor heterogeneity of HBV-associated hepatocellular carcinoma. Nat Commun.

[184] Zheng C, Zheng L, Yoo JK, Guo H, Zhang Y, Guo X, Kang B, Hu R, Huang JY, Zhang Q (2017). Landscape of infiltrating T cells in liver cancer revealed by single-cell sequencing. Cell.

[185] Xiang B, Deng C, Qiu F, Li J, Li S, Zhang H, Lin X, Huang Y, Zhou Y, Su J (2021). Single cell sequencing analysis identifies genetics-modulated ORMDL3(+) cholangiocytes having higher metabolic effects on primary biliary cholangitis. J Nanobiotechnol.

[186] Abdel-Hakeem MS, Manne S, Beltra JC, Stelekati E, Chen Z, Nzingha K, Ali MA, Johnson JL, Giles JR, Mathew D (2021). Epigenetic scarring of exhausted T cells hinders memory differentiation upon eliminating chronic antigenic stimulation. Nat Immunol.

[187] Keener AB (2019). Single-cell sequencing edges into clinical trials. Nat Med.

[188] Kashif M, Alici E, Nahi H (2021). Predicting drug resistance by single-cell RNASeq in patients with multiple myeloma. Clin Chem.

[189] Cohen YC, Zada M, Wang S-Y, Bentur OS, Chubar E, Cohen A, Lavi N, Magen H, Gatt M, Zektser M (2021). Single cell RNA sequencing in patients enrolled in a selinexor clinical trial reveals overexpression of alternative nuclear export pathways associated with resistance to selinexor in refractory multiple myeloma. Blood.

[190] Shi Z, Zhang Q, Yan H, Yang Y, Wang P, Zhang Y, Deng Z, Yu M, Zhou W, Wang Q (2019). More than one antibody of individual B cells revealed by single-cell immune profiling. Cell Discov.

