# Supplementary material for: Liver in infections: a single-cell and spatial transcriptomics perspective
Source: J Biomed Sci. 2023 Jul 10;30:53. doi: 10.1186/s12929-023-00945-z (PMC10332047; doi:10.1186/s12929-023-00945-z)
Supplement: Supplementary file 1 — Additional file 1: Figure S1. Single cell perspective of liver in HBV infection. A HBV maintenance in hepatocytes. The structure diagram of NTCP is quoted from REF [160]. B An unbiased and comprehensive landscape of the intrahepatic immunological characteristic in HBV-infected patients. C The roles of Kuffer cellsin HBV-infection. D A systematic and distinguishing gene regulatory network of CHB-related PBMCs. E The immune microenvironment of infected liver in FVH. Figure S2. Clinical implication of high throughout single cell technology. [file 12929_2023_945_MOESM1_ESM.docx]

**Additional figures and legends**


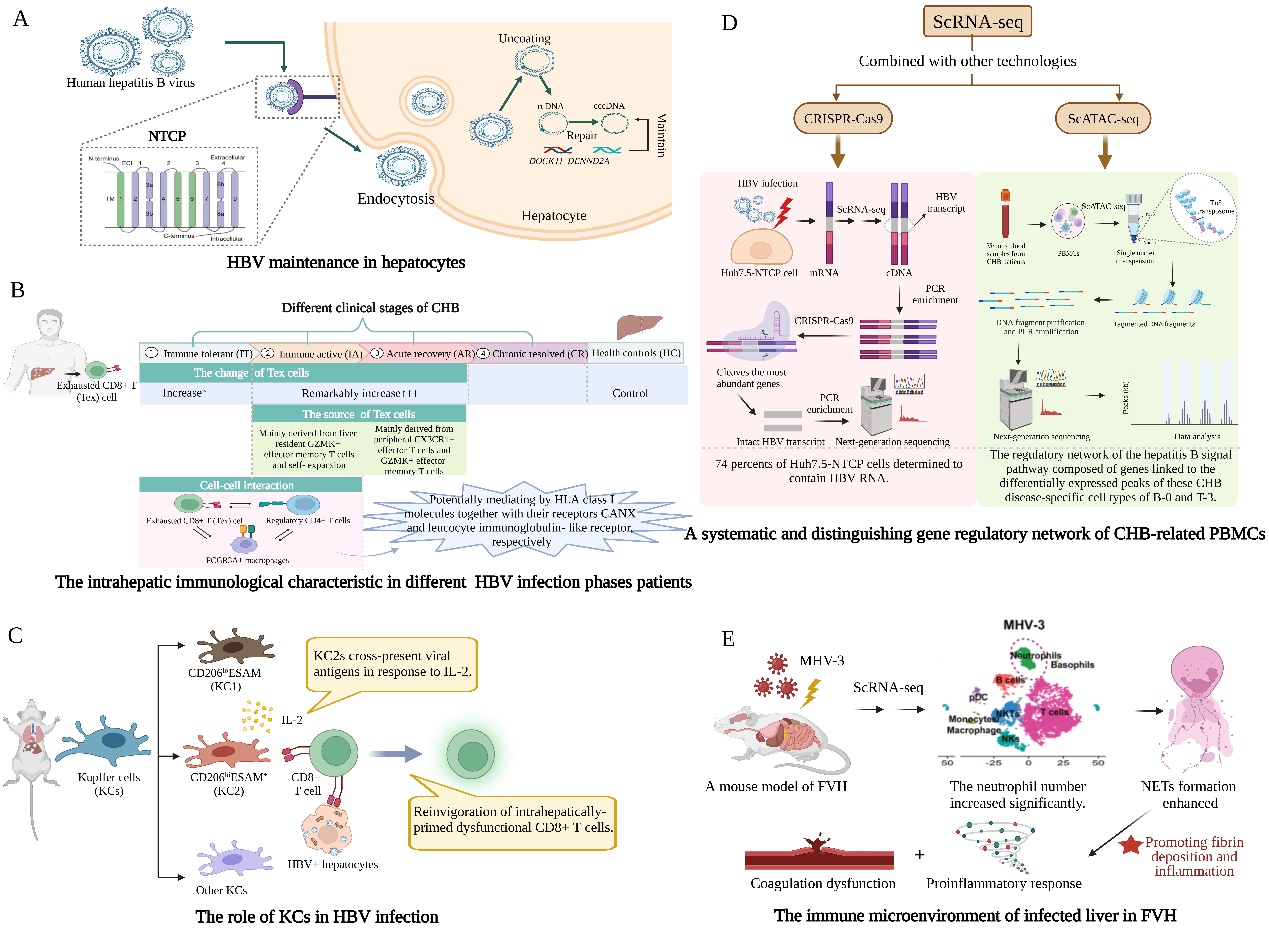


**Figure S1: Single cell perspective of liver in HBV infection. (A)**HBV maintenance in hepatocytes. The structure diagram of NTCP is quoted from REF 172. **(B)** An unbiased and comprehensive landscape of the intrahepatic immunological characteristic in HBV-infected patients **(C)** The roles of Kuffer cells (KCs) in HBV-infection **(D)** A systematic and distinguishing gene regulatory network of CHB-related PBMCs **(E)** The immune microenvironment of infected liver in FVH.

**Abbreviation:** AR, acute recovery; cccDNA, covalently closed circular DNA; CHB, chronic Hepatitis B; CR, chronic resolved; CRISPR, clustered regularly interspaced short palindromic repeats; CRISPR-Cas9，CRISPR-CRISPR-associated nuclease 9; FVH, fulminant viral hepatitis; HCC, hepatocellular carcinoma; HBV, hepatitis B Virus; HC, health controls; HLA, human leukocyte antigen; IL-2, interleukin-2; IT, immune tolerant; IA, immune active; KC, kupffer cell; MHV-3, murine hepatitis virus strain-3; NTCP, Na^+^-taurocholate cotransporting polypeptide; NETs, neutrophil extracellular traps; PCR, polymerase chain reaction; rcDNA, relaxed circular DNA; ScRNA-seq, single-cell RNA sequencing; ScATAC-seq, single-cell assay for transposase-accessible chromatin using sequencing.


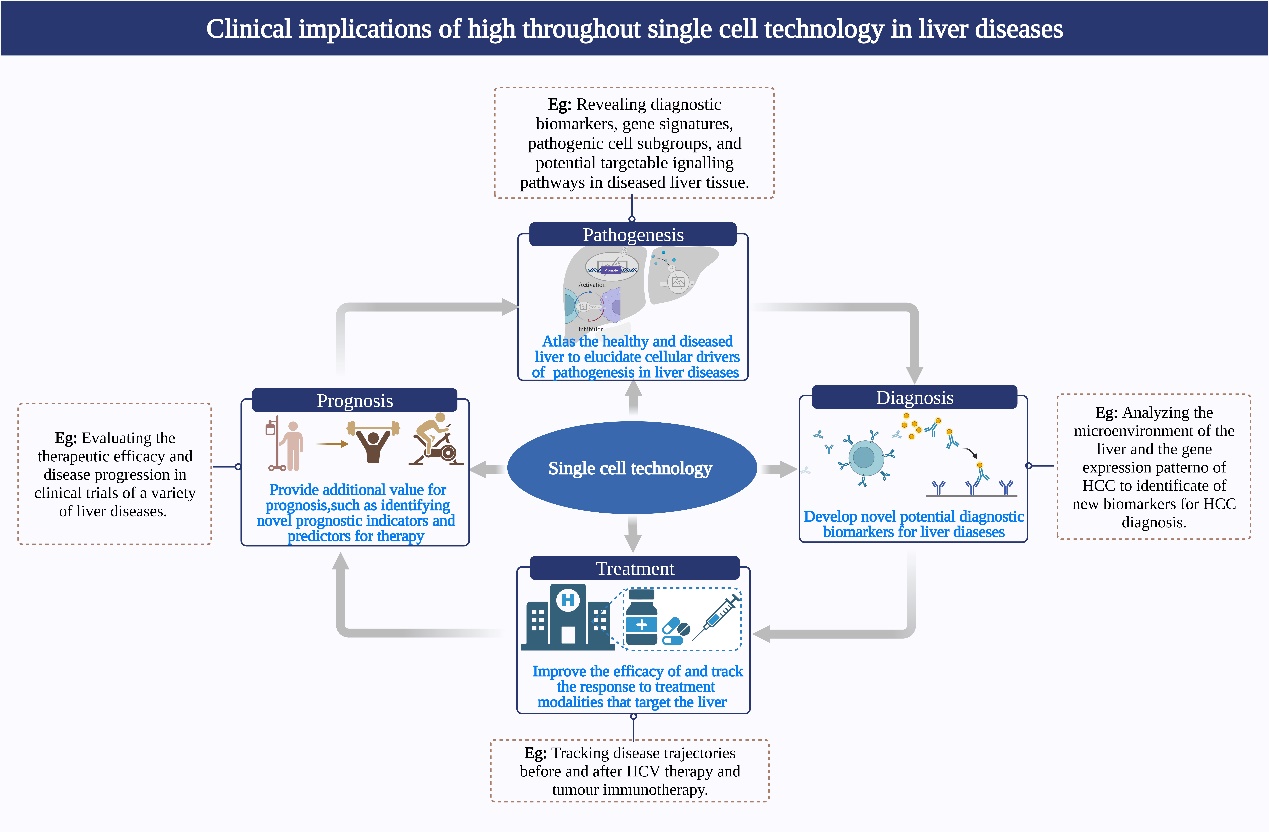


**Figure S2: Clinical implication of high throughout single cell technology.**
